# A feedback loop between *CaWRKY41* and H_2_O_2_ coordinates the response to *Ralstonia solanacearum* and excess cadmium in pepper

**DOI:** 10.1093/jxb/erz006

**Published:** 2019-01-14

**Authors:** Fengfeng Dang, Jinhui Lin, Yongping Chen, Gui Xin Li, Deyi Guan, Shao Jian Zheng, Shuilin He

**Affiliations:** 1 State Key Laboratory of Plant Physiology and Biochemistry, College of Life Sciences, Zhejiang University, Hangzhou, China; 2 Key Laboratory of Plant Genetic Improvement and Comprehensive Utilization of the Ministry of Education, College of Crop Science, Fujian Agriculture and Forestry University, Fuzhou, Fujian, China; 3 College of Horticulture, Fujian Agriculture and Forestry University, Fuzhou, Fujian, China; 4 College of Agronomy and Biotechnology, Zhejiang University, Hangzhou, China

**Keywords:** *Capsicum annuum*, *CaWRKY41*, cadmium, H_2_O_2_, *Ralstonia solanacearum*, reactive oxygen species

## Abstract

WRKY transcription factors have been implicated in both plant immunity and plant responses to cadmium (Cd); however, the mechanism underlying the crosstalk between these processes is unclear. Here, we characterized the roles of CaWRKY41, a group III WRKY transcription factor, in immunity against the pathogenic bacterium *Ralstonia solanacearum* and Cd stress responses in pepper (*Capsicum annuum*). *CaWRKY41* was transcriptionally up-regulated in response to Cd exposure, *R. solanacearum* inoculation, and H_2_O_2_ treatment. Virus-induced silencing of *CaWRKY41* increased Cd tolerance and *R. solanacearum* susceptibility, while heterologous overexpression of *CaWRKY41* in Arabidopsis impaired Cd tolerance, and enhanced Cd and zinc (Zn) uptake and H_2_O_2_ accumulation. Genes encoding reactive oxygen species-scavenging enzymes were down-regulated in *CaWRKY41*-overexpressing Arabidopsis plants, whereas genes encoding Zn transporters and enzymes involved in H_2_O_2_ production were up-regulated. Consistent with these findings, the *ocp3* (*overexpressor of cationic peroxidase 3*) mutant, which has elevated H_2_O_2_ levels, displayed enhanced sensitivity to Cd stress. These results suggest that a positive feedback loop between H_2_O_2_ accumulation and *CaWRKY41* up-regulation coordinates the responses of pepper to *R. solanacearum* inoculation and Cd exposure. This mechanism might reduce Cd tolerance by increasing Cd uptake via Zn transporters, while enhancing resistance to *R. solanacearum*.

## Teaser

*CaWRKY41* plays dual roles in increasing resistance to *Ralstonia solanacearum* infection and susceptibility to Cd stress in pepper by indirectly and directly increasing H_2_O_2_ accumulation and Zn transporter expression.

## Introduction

Plants are frequently exposed to various biotic and abiotic stresses in their natural habitats. A variety of defense response mechanisms have evolved that protect the plant against particular stresses. These mechanisms are mediated by complex signaling pathways, which must be coordinately and tightly regulated. Common signaling pathways such as MAPK cascades (Rodriguez *et al.*, 2010; Meng and Zhang, 2013) and pathways involving calcium (Knight, 2000; Bose *et al.*, 2011) and reactive oxygen species (ROS; (Qi *et al.*, 2017) are ubiquitously involved in plant responses to various biotic or abiotic stresses, suggesting that they coordinate these responses. However, the exact roles of most of these signaling components and how they are functionally linked are poorly understood.

ROS, including the superoxide radical (O_2_^−^), hydrogen peroxide (H_2_O_2_), hydroxyl radical (OH^•^), and singlet oxygen (^1^O_2_), are partially reduced forms of molecular oxygen (O_2_) that typically result from the transfer of one, two, or three electrons to O_2_. H_2_O_2_ is the most stable ROS, with a relatively long half-life (~1 ms in the cell), and often acts as an intercellular and intracellular signal that triggers downstream responses (Baxter *et al.*, 2014; Camejo *et al.*, 2016). ROS homeostasis is modulated by various enzymes; ROS production in multiple subcellular locations is associated with the activities of NADPH oxidases [or respiratory burst oxidase homologs (RBOHs)], glycolate oxidases, and peroxidases (Mittler, 2002; Suzuki *et al.*, 2011; Marino *et al.*, 2012; Gupta *et al.*, 2017). ROS are scavenged by the antioxidant system, including non-enzymatic antioxidants such as ascorbic acid and glutathione, and several antioxidant enzymes, such as catalase (CAT), ascorbate peroxidase (APX), monodehydroascorbate reductase, dehydroascorbate reductase, glutathione reductase, glutathione peroxidase, and glutathione-*S*-transferase (Romero-Puertas *et al.*, 2007; Dinakar *et al.*, 2010). The production and decomposition of ROS are balanced under non-stress conditions. However, under various environmental stress conditions, this balance frequently breaks down, resulting in a burst of ROS (Lv *et al.*, 2017). Although excess ROS cause oxidative injury, these molecules also act as second messengers that regulate physiological and developmental processes in plants under both stress and non-stress conditions (Apel and Hirt, 2004; Baxter *et al.*, 2014; Qi *et al.*, 2017).

Accumulating evidence indicates that ROS bursts are crucial regulators of plant immunity (Torres *et al.*, 2006; Mersmann *et al.*, 2010; Vellosillo *et al.*, 2010). The perception of pathogen-associated molecular patterns by pattern recognition receptors, and of specific pathogen effectors (either directly or indirectly) by specific nucleotide-binding leucine-rich repeat receptors, triggers ROS bursts in the plant through the activation of RBOHs and peroxidases (Schwizer *et al.*, 2017). ROS bursts are thought to reinforce the cell wall around points of infection and activate downstream responses including defense gene expression, the production of antimicrobial compounds, and the hypersensitive response (Alvarez *et al.*, 1998; Torres *et al.*, 2006). Virulent pathogens possess effectors that are capable of suppressing ROS bursts in various ways and thereby suppressing downstream immune responses during infection (Shidore *et al.*, 2017). Thus, ROS may act as overlapping components in pathogen-associated molecular pattern-triggered immunity and effector-triggered immunity, and serve as crucial nodes connecting these processes (Tsuda and Katagiri, 2010; Adachi *et al.*, 2015).

ROS bursts are also a primary effect of exposure to excess cadmium (Cd). This element, which is released into the agricultural ecological system as a result of urbanization and industrialization, is considered to be one of the most toxic heavy metals in the environment (Gupta *et al.*, 2017). Cd is thought to induce the formation of ROS indirectly by inhibiting the activity of antioxidant enzymes, impairing the respiratory chain, or displacing copper and iron ions from metalloenzymes and interfering with the redox status of the cell (Valko *et al.*, 2005). ROS production in response to Cd exposure may cause oxidative injury to plants, but the exact roles of ROS in the plant response to Cd exposure are poorly understood. As ROS are associated with the plant response to pathogen infection and Cd toxicity, these processes are thought to be linked via ROS. Indeed, treatment with salicylic acid (SA), a defense-signaling molecule, alleviates Cd toxicity in barley (*Hordeum vulgare*) seedlings (Metwally *et al.*, 2003). Moreover, Cd concentrations close to the toxicity threshold induce defense-signaling pathways mediated by SA and jasmonic acid (Cabot *et al.*, 2013). However, the exact roles of ROS in plant responses to Cd tolerance, and whether and how plant immunity and responses to Cd stress are coordinated by ROS, remain to be elucidated.

A key step in plant responses to diverse stresses is the transcriptional reprogramming of a multitude of defense-associated genes by various transcription factors (TFs). WRKY proteins, which are characterized by the presence of one or two highly conserved WRKY domains, constitute one of the largest TF families. WRKY TFs are important positive and negative regulators of plant growth and development, and of defense responses to environmental stimuli (Eulgem *et al.*, 2000; Rushton *et al.*, 2010). While this large family of TFs is mainly involved in regulating plant immune responses (Sarris *et al.*, 2015), a few WRKY members, including *Tamarix hispida* WRKY7 (Yang *et al.*, 2016) and *Zea mays* WRKY4 (Hong *et al.*, 2017), have been implicated in plant responses to Cd toxicity. In addition, some WRKY TFs are involved in more than one biological process, suggesting that WRKYs are crucial nodes in the crosstalk between plant immunity and other biological processes (Rushton *et al.*, 2010). Moreover, the expression of most group III *WRKY* genes is modified in response to pathogen attack and treatment with SA (Kalde *et al.*, 2003). As recent studies have shown that group III *WRKY* genes play important roles in plant responses to abiotic stress (Li *et al.*, 2013; Ding *et al.*, 2014; Chen *et al.*, 2017), we reasoned that these genes might be involved in the crosstalk between plant responses to pathogen attack and abiotic stress, possibly coordinating plant responses to these stresses.

Pepper (*Capsicum annuum*) is a solanaceous vegetable crop widely grown around the world. Blight and bacterial wilt caused by the soil-borne pathogens *Phytophthora capsici* and *Ralstonia solanacearum*, respectively, frequently reduce pepper production. Heavy metal contamination is another factor that inhibits pepper growth. Heavy metal residues are present in soils as a result of sewage irrigation and the use of heavy-metal-containing products such as pesticides and fertilizers. A better understanding of how pepper responds to heavy metal contamination would lay the foundations for developing effective countermeasures.

In the present study, we investigated the transcriptional responses of group III *WRKY*s to Cd toxicity and *R. solanacearum* inoculation. We also investigated the responses of these genes to iron (Fe) deficiency, because Cd toxicity-induced chlorosis resembles Fe deficiency-induced chlorosis (Sun *et al.*, 2015; Chen *et al.*, 2016; Li *et al.*, 2016), and plant responses to Fe deficiency are related to responses to excess Cd (Nakanishi *et al.*, 2006; Han *et al.*, 2014; Mendoza-Cozatl *et al*., 2014). Among the eight group III *WRKY* genes we examined, only *CaWRKY41* was synergistically up-regulated in pepper plants challenged by Cd toxicity, Fe deficiency, or *R. solanacearum* inoculation. We identified a positive feedback loop between *CaWRKY41* and H_2_O_2_ accumulation during the response to *R. solanacearum* inoculation and excess Cd exposure in pepper.

## Materials and methods

### Plant materials and growth conditions

Seeds of pepper (*Capsicum annuum*) 8# (an inbred line provided by the pepper breeding group at Fujian Agriculture and Forestry University) and CM334 (Mexican landrace of *C. annuum* cv*. CM334*), and tobacco (*Nicotiana benthamiana*) were imbibed in sterile water at 25±2 °C overnight and sown in a steam-sterilized soil mix (peat moss, vermiculite, and perlite, 2:1:1 by volume) in plastic pots.

Pepper plants were grown in a growth room maintained at 25±2 °C with a light intensity of ~100 µmol photons m^−2^ s^−1^ and a relative humidity of 70% under a 16 h light/8 h dark cycle. For liquid cultivation, 21-day-old pepper seedlings were transferred to 1.2 l black plastic beakers containing modified one-fifth Hoagland solution. The initial nutrient solution contained the macronutrients KNO_3_ (1 mM), Ca (NO_3_)_2_·4H_2_O (1 mM), MgSO_4_·7H_2_O (1.4 mM), and KH_2_PO_4_ (0.2 mM), and the micronutrients Fe-EDTA (20 µM), H_3_BO_3_ (3 µM), (NH_4_)_6_Mo_7_O_24_ (1 µM), MnCl_2_ (0.5 µM), ZnSO_4_ (0.4 µM), and CuSO_4_ (0.2 µM). The pH of the solution was adjusted to 5.8, and the nutrient solution was renewed every 3 days.

For *Arabidopsis thaliana* cultivation, wild-type (WT; Col-0), *ocp3* (Coego *et al.*, 2005), *CaWRKY41-OE1*, and *CaWRKY41-OE4* transgenic Arabidopsis seeds were treated by exposure to 4 °C in darkness for 3 days and then sown on vertically placed Petri dishes containing ½ Murashige and Skoog (MS) medium (PhytoTechnology, product ID M524) supplemented with 1% (w/v) sucrose and 0.8% agar (Sigma, cat. no. A1296) in the absence or presence of heavy metals or other supplements in a growth chamber maintained at 22±2 °C with a light intensity of ~100 µmol photons m^−2^ s^−1^ and a relative humidity of 70%, under a 16 h light/8 h dark cycle.

### Phylogenetic analysis of group III WRKY TFs across three plant species

The WRKY TFs were described previously (Eulgem *et al.*, 2000). The amino acid sequences of proteins and domains of group III CaWRKYs, SlWRKYs, and AtWRKYs from the *C. annuum*, *Solanum lycopersicum*, and *A. thaliana* genomes were downloaded from Plant TFDB V4.0 (http://planttfdb.cbi.pku.edu.cn/index.php).

### Pathogens and inoculation procedures

*Ralstonia solanacearum* strain FJ150501 was isolated from pepper plants showing symptoms of bacterial wilt infection in Guangdong Province, China. For soil-drenching inoculation, *PYL-279* and *PYL-279-wrky41* pepper plants grown in pots, with the roots partially and mechanically damaged, were inoculated with a 10^8^ cfu/ml (OD_600_=0.8) suspension of *R. solanacearum*. A disease index (from 0 to 5) was scored daily in the *R. solanacearum-*inoculated pepper plants, as follows: 0 (no wilting), 1 (1 to 20% wilted), 2 (21 to 40% wilted), 3 (41 to 60% wilted), 4 (61 to 80% wilted), and 5 (81 to 100% wilted or dead). The average values reported are based on three independent replicates, each comprising six plants. Electrolyte leakage was measured in pepper leaves at 0, 24, and 48 h post infection. For suspension inoculation, pepper plants were grown in 1.2 l black plastic beakers containing one-fifth Hoagland solution. Eight of the lateral roots were removed from each plant with a pair of scissors, and the plants were then transferred to one-fifth Hoagland solution with 10^8^ cfu/ml *R. solanacearum* suspension.

### Plasmid construction and plant transformation

To construct the vector *35S::CaWRKY41*, the full-length open reading frame was cloned into pDONR207 and transferred into the pGWB2 expression vector (Invitrogen, USA). To construct the reporter vector (*pCaWRKY41::GUS*) for histochemical β-glucuronidase (GUS) analysis, the promoter of *CaWRKY41* of ~2 kb in length (*pCaWRKY41*) was amplified via PCR from pepper genomic DNA and cloned into the pMDC163 vector (Invitrogen). The constructs *35S::CaWRKY41* and *pCaWRKY41::GUS* were then transformed into *Agrobacterium tumefaciens* strain GV3101 using the freeze–thaw method. *A. tumefaciens*-mediated transformation of Arabidopsis was performed using the floral dip method (Clough and Bent, 1998), and transgenic plants were identified by sowing seeds on ½ MS agar plates containing 50 mg l^−1^ hygromycin and selecting hygromycin-resistant seedlings.

### Subcellular localization and transcriptional activity analysis

The coding region of *CaWRKY41* without the stop codon was cloned into the *pCambia1300-GFP/C* vector by In-Fusion Cloning (Clontech, USA). The *pCambia1300-CaWRKY41-GFP* construct was transformed into *A. tumefaciens* GV3101 and infiltrated into the fully expanded leaves of 5-week-old *N. benthamiana* plants. At 2 days post inoculation, green fluorescent protein (GFP) fluorescence was observed by confocal laser-scanning microscopy (Zeiss LSM710, Germany). For the transactivation assay, the open reading frames of *CaWRKY41* (1–329) and the mutant genes *CaWRKY41* (61–329), *CaWRKY41* (131–329), and *CaWRKY41* (192–329) were generated by PCR with specific primer pairs and cloned into *pGBKT7* (Clontech) to generate various *CaWRKY41* constructs (BD-*CaWRKY41*,-*1*,-*2* and -*3*). Transcriptional activation activity was determined in yeast cells transformed with these constructs grown on SD medium lacking Trp for 3 days, and a colony-lift filter assay (X-gal assay) was performed.

### Virus-induced gene silencing

*CaWRKY41*-silenced pepper plants were generated using tobacco rattle virus-based virus-induced gene silencing (VIGS) as described previously (Dang *et al.*, 2013). Briefly, a specific 328 bp fragment of *CaWRKY41* was identified by homologous searching via BLAST analysis against the genome sequences of pepper cultivars CM334 (http://peppergenome.snu.ac.kr/) and Zunla-1 (http://peppersequence.genomics.cn/page/species/blast.jsp). The fragment was cloned into the entry vector *pDONR207* and then into the *PYL279* vector. The vectors (*PYL-279* and *PYL-279-wrky41*) were separately transformed into *A. tumefaciens* GV3101 cells, which were subsequently mixed with *A. tumefaciens* cells harboring *PYL-192* and injected into fully expanded pepper seedling cotyledons. *PYL-279-wrky41* pepper plants were subjected to experimental analysis, with *PYL-279* plants (transformed with empty vector) serving as a control. Levels of H_2_O_2_ and of the expression of various genes were measured in *CaWRKY41-*silenced *PYL-279* and *PYL-279-wrky41* pepper plants grown in liquid culture.

### Treatment of plants with Cd and exogenous application of H_2_O_2_

To test the effect of Cd on seed germination and growth in Arabidopsis, seeds were treated by exposure to 4 °C in darkness for 3 days and then grown on ½ MS medium containing 25 µM, 50 µM, or 100 µM CdSO_4_ for 8 days. To measure the expression of various genes in plants in the presence of excess Cd supply, 7-day-old Arabidopsis seedlings were transferred to ½ MS medium without or with 25 µM CdSO_4_, cultured for 6 or 72 h, and harvested for use. To investigate the expression of the eight *CaWRKY* group III genes, pepper plants at the six-leaf stage grown in liquid culture were treated with Cd stress (2.5, 5, 25, and 60 µM CdSO_4_) and Fe deficiency (0 µM Fe-EDTA). Pepper plants at the six-leaf stage were sprayed with H_2_O_2_ (1 mM) and incubated for 0, 1, 3, 6, 12, 24, 36, and 48 h, and leaf tissue was harvested for *CaWRKY41* expression analysis.

### Histochemical staining

Leaves were stained with Trypan blue and 3, 3′-diaminobenzidine (DAB) as described previously (Dang *et al.*, 2013, 2014; Cai *et al.*, 2015). For GUS staining, seedlings or tissues were incubated overnight in GUS staining solution (1 mg·ml^−1^ X-Gluc, 1 mM K_3_Fe(CN)_6_, 1 mM K_4_Fe(CN)_6_, 50 mM sodium phosphate buffer pH 7.0, 10 mM Na_2_EDTA, and 0.1% Triton X-100) at 37 °C, destained several times in 75% (v/v) ethanol, and observed under a stereomicroscope (Leica, Germany).

### Measurement of H_2_O_2_ and Cd contents and enzyme activity

Seedlings were grown on ½ MS medium for 7 days, treated with 25 µM CdSO_4_ for 3 and 5 days, and sampled for H_2_O_2_ and Cd analysis and enzymatic assays. For H_2_O_2_ measurements, seedlings were harvested, ground in liquid nitrogen, and examined using an Amplex Red H_2_O_2_-peroxidase Assay Kit (Molecular Probes). This one-step assay uses Amplex Red reagent (10-acetyl-3,7-dihydroxyphenoxazine) to detect H_2_O_2_. Briefly, approximately 80 mg of sample was processed and measured using an H_2_O_2_ standard curve. The fluorescence emission spectrum (590 nm) was detected at an excitation wavelength of 530 nm using a Tecan Infinite 200 Pro (Tecan, Switzerland).

To measure the Cd contents in roots and shoots, the roots were rinsed three times (for 4 min each time) with Milli-Q water to remove Cd attached to the root surface. The root and shoot samples were weighed and digested with 0.5 ml (for roots) and 1 ml (for shoots) concentrated HNO_3_. Each sample was adjusted to 10 ml with Milli-Q water and then filtered through filter paper. Cd in the samples was detected by inductively coupled plasma-atomic emission spectrometry (IRIS/AP Optical Emission Spectrometer, Thermo Scientific, USA). The experiment was performed in three biological replicates.

For enzymatic activity analysis, approximately 80 mg of sample was ground in liquid nitrogen using a TissueLyserII, and milled samples were homogenized in phosphate buffer (600 μl, 50 mM, pH 7.0) and centrifuged at 3000 × *g* at 4 °C for 10 min. Then, peroxidase (POD), CAT, and APX activity were analyzed using an ELISA kit (Shanghai Bangyi Biotechnology Co. Ltd, China) according to the manufacturer’s instructions. Microtiter plate wells were coated with purified POD, CAT, and APX antibody, to make a solid-phase antibody, and then samples were added to the wells together with an antibody labeled with horseradish peroxidase, and an antibody–antigen–enzyme complex formed. Substrate solution was added after thorough washing, and then, using a blank well as the zero control, the absorbance was measured at 450 nm in a Tecan Infinite 200 Pro Plate Reader (Tecan).

### RNA extraction and reverse transcription–quantitative PCR (RT–qPCR)

Total RNA was isolated from Arabidopsis and pepper tissues using a TaKaRa Mini BEST Universal RNA Extraction Kit (TaKaRa, Dalian, China). RNA (1 µg) was used as a template to synthesize cDNA with a TaKaRa PrimeScript RT-PCR Kit (TaKaRa) according to the manufacturer’s instructions. Gene expression levels were measured on a CFX96 Real-Time PCR System (Bio-Rad, USA) using SYBR^®^ Premix Ex Taq™ II (TaKaRa); specific primers are listed in Supplementary Table S1 at *JXB* online. Arabidopsis *UBIQUITIN10* (*AtUBQ10*) and pepper *Actin1* (*CaActin1*) were used for normalization.

## Results

### Phylogenetic analysis of *CaWRKY* group III genes

To identify the phylogenetic relationships among the eight *CaWRKY* group III genes, we compared their nucleotide sequences to those of WRKY genes from tomato and Arabidopsis. We constructed an unrooted phylogenetic tree based on an alignment of the amino acid sequences of the group III WRKY proteins and domains from the three plant species using the neighbor-joining method. Based on this analysis, WRKYs from pepper share higher sequence similarity with WRKYs from tomato than with those from *Arabidopsis* (see Supplementary Fig. S1). Detailed information about the *CaWRKY* group III genes is provided in Supplementary Tables S2 and S3.

### Expression analysis of eight *CaWRKY* group III genes during exposure to excess Cd or Fe deficiency

Cd is a highly toxic heavy metal that is readily absorbed by plant roots, loaded into the xylem, and transported to leaves, leading to the generation of ROS (Valko *et al.*, 2005; Perez-Chaca *et al.*, 2014; Keunen *et al.*, 2015). ROS production has been detected in sunflower (*Helianthus annuus L.*) and maize (*Z. mays*) under conditions of Fe deficiency (Ranieri *et al.*, 2001; Sun *et al.*, 2007).

**Fig. 1.**
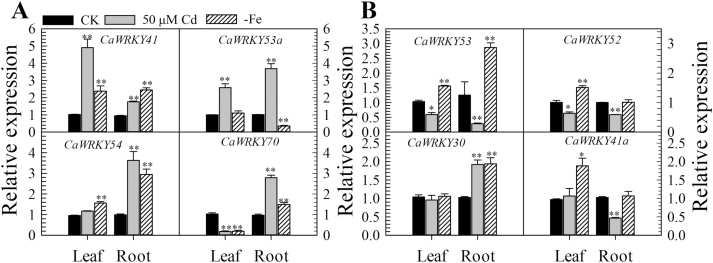
(A, B) Expression of eight group III *WRKY* genes in the leaves and roots of pepper plants after 24 h of exposure to Cd stress and Fe deficiency, as determined by RT–qPCR analysis. The relative expression of the genes in stressed plants was compared with that of control untreated (CK) plants, which was set to a value of 1. Data represent the mean ±SE of three biological replicates. Asterisks indicate significant differences compared with CK plants (Student’s *t-*test; *P<0.05, **P<0.01).

In the present study, H_2_O_2_ accumulation was detected in DAB-stained pepper leaves after 24, 36, and 48 h of Cd stress and Fe deficiency treatments (see Supplementary Fig. S2A, B). Similar to the response to Cd stress, the newly emerged leaves of pepper plants at the eight-leaf stage displayed yellowing after Fe deficiency treatment (Supplementary Fig. S2C, D). To identify the group III WRKY TFs involved in Cd stress, we measured the expression of the eight group III *WRKY* genes by RT–qPCR analysis in pepper plants exposed to Cd stress or Fe deficiency. *CaWRKY41* and *CaWRKY53a* expression significantly increased under Cd stress in both the roots and leaves of pepper plants (Fig. 1A). Furthermore, *CaWRKY41*, *CaWRKY53*, and *CaWRKY54* expression markedly increased under Fe deficiency treatment in both roots and leaves (Fig. 1). Therefore, among the eight group III WRKY genes in pepper, only *CaWRKY41* expression was up-regulated by both Cd toxicity and Fe deficiency in roots and leaves, pointing to the involvement of CaWRKY41 in the response of pepper to excess Cd and Fe deficiency, which might be associated with the production of H_2_O_2_.

### *CaWRKY41* is up-regulated in response to Cd and H_2_O_2_

**Fig. 2.**
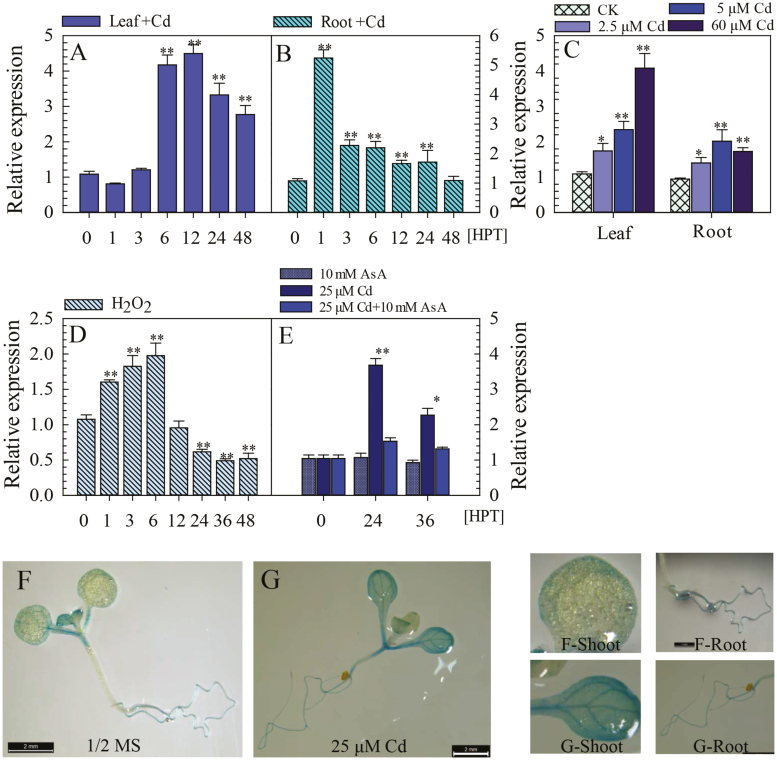
*CaWRKY41* was transcriptionally induced by Cd and H_2_O_2_ treatment in pepper. (A, B) *CaWRKY41* expression in pepper leaves and roots determined by RT–qPCR at the indicated time points after treatment with 25 µM CdSO_4_. HPT, hours post treatment. (C) *CaWRKY41* expression in pepper leaves and roots determined by RT–qPCR at 12 HPT with 2.5, 5, or 60 µM CdSO_4_. CK, control untreated. (D) *CaWRKY41* expression in pepper leaves analyzed at 0, 1, 3, 6, 12, 24, 36, and 48 HPT with 1 mM H_2_O_2_. Relative expression levels of *CaWRKY41* in stressed plants were compared with those of control plants, which were set to a value of 1. Data represent the mean ±SE of three biological replicates. Asterisks indicate significant differences compared with control plants (Student’s *t-*test; *P<0.05, **P<0.01). (E) Excess Cd-induced expression of *CaWRKY41* in pepper leaves was inhibited by treatment with 10 mM ascorbic acid (AsA). (F, G) GUS expression in transgenic Arabidopsis plants carrying the *pCaWRKY41::GUS* construct. Seven-day-old *pCaWRKY41::GUS* seedlings were transferred to ½ MS medium without (F) or with (G) 25 µM CdSO_4_ for 12 h, followed by staining. Panels labeled F-shoot, F-root, G-shoot, and G-root show magnifications of the corresponding plant parts shown in panel F or G, respectively, to show details of the GUS staining patterns of shoots and roots of *pCaWRKY41::GUS* seedlings. Plants were grown on ½ MS medium under 16 h light/8 h dark conditions.

To further investigate the involvement of CaWRKY41 in the response of pepper to Cd toxicity, we measured the time course and dose-responsive patterns of *CaWRKY41* expression in response to Cd stress by RT–qPCR analysis. After exposure to excess Cd, *CaWRKY41* expression was strongly enhanced, peaking at 12 h post treatment (HPT) in the leaves and 1 HPT in the roots (Fig. 2A, B). *CaWRKY41* expression was also increased in response to treatment with 2.5, 5, and 60 µM Cd compared with the control (Fig. 2C). Additionally, *CaWRKY41* expression was significantly up-regulated in response to exogenous application of H_2_O_2_ (Fig. 2D). However, the *CaWRKY41* expression in pepper leaves triggered by excess Cd was reduced when samples were treated with the H_2_O_2_ scavenger ascorbic acid (Fig. 2E, Supplementary Fig. S2E).

To confirm the expression pattern of *CaWRKY41*, we generated *pCaWRKY41::GUS* transgenic Arabidopsis plants. Seven-day-old *pCaWRKY41::GUS* seedlings were transferred to ½ MS medium without or with excess Cd for 12 h and then stained to analyze GUS activity. When *pCaWRKY41::GUS* seedlings were transferred to conditions of excess Cd, increased GUS activity was observed in the shoot and root (Fig. 2F, G). When *pCaWRKY41::GUS* seedlings were grown under normal conditions, GUS staining was consistently detected in the roots, shoots, mature leaves, and petioles (Supplementary Fig. S3A–G). Intensive GUS staining was also observed in the flowers (Supplementary Fig. S3H, I) but not in the siliques (Supplementary Fig. S3J). These results imply that *CaWRKY41* might be involved in the response of pepper to excess Cd and H_2_O_2_ accumulation.

### Analysis of the subcellular localization and transcriptional activity of CaWRKY41

As the function of a given protein is closely related to its subcellular localization, we investigated the subcellular localization of CaWRKY41 in transiently transformed *N. benthamiana* leaves harboring the open reading frame of this gene, without the translation terminator, driven by the *35S* promoter and fused to the *GFP* gene. The CaWRKY41-GFP fusion protein was exclusively localized to the nuclei of epidermal cells when heterologously expressed in *N. benthamiana* (Supplementary Figs S3K and S4A).

In addition, we assayed the transcriptional activity of CaWRKY41 in yeast via a transcriptional activation assay. The expression of the *LacZ* reporter gene driven by the GAL4 upstream activating sequence was significantly increased by the presence of the BD-CaWRKY41 fusion protein in yeast, but *LacZ* expression was not induced in the negative control (Supplementary Fig. S4B). These results indicate that CaWRKY41 is a nuclear protein with transcriptional activity.

### *CaWRKY41* silencing increases Cd tolerance and reduces H_2_O_2_ accumulation in pepper

**Fig. 3.**
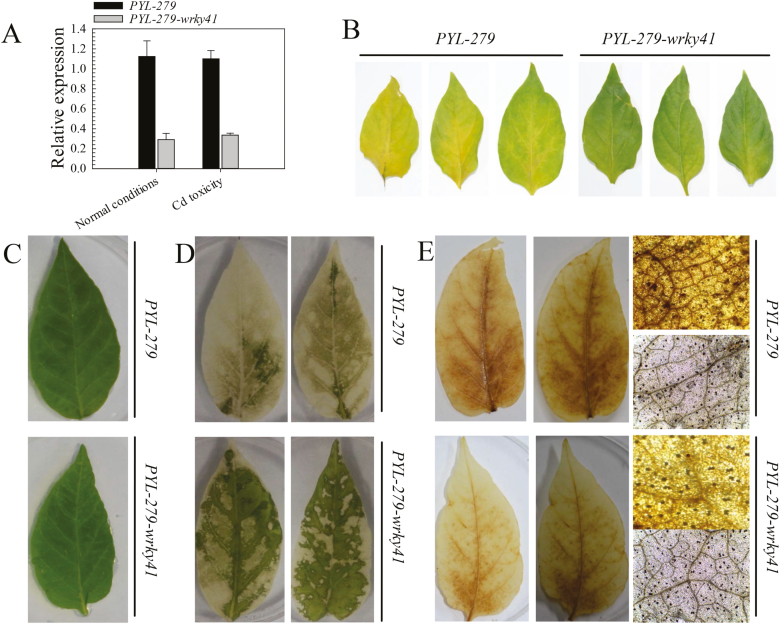
*CaWRKY41* silencing enhances tolerance to Cd stress in pepper. (A) *CaWRKY41* expression in *PYL-279* (control) and *PYL-279-wrky41* pepper leaves. (B) Less yellowing was observed in *PYL-279-wrky41* compared with *PYL-279* pepper leaves. Pepper plants were grown in one-fifth Hoagland solution. When photobleaching was observed in *PYL-279-pds* leaves, *PYL-279-wrky41* and *PYL-279* plants were transferred to fresh nutrient solution containing 50 µM CdSO_4_ for 4 days. (C, D) Leaves from *PYL-279* and *PYL-279-wrky41* cultured on 1/5 MS medium without (C) or with (D) 25 µM CdSO_4_ for 4 days. (E) H_2_O_2_ production observed after 3, 3′-diaminobenzidine staining in leaves of *PYL-279* and *PYL-279-wrky41* plants at 3 days post treatment with 25 µM CdSO_4_.

The induction of *CaWRKY41* expression by excess Cd points to its involvement in the Cd stress response. To test this possibility, we examined the effect of VIGS of *CaWRKY41* on the response of pepper to Cd stress. To avoid possible off-target silencing, we inserted a specific 328 bp fragment of *CaWRKY41* into the *PYL-279-wrky41* vector [tobacco rattle virus (*PYL-279*): *wrky41*] to silence *CaWRKY41* in pepper. *CaWRKY41* was expressed at a level approximately 3.8- and 3.2-fold lower in *CaWRKY41*-silenced plants than in control plants (*PYL-279*), in the presence and absence of Cd stress, respectively (Fig. 3A), respectively, indicating that we had successfully silenced *CaWRKY41* via VIGS.

**Fig. 4.**
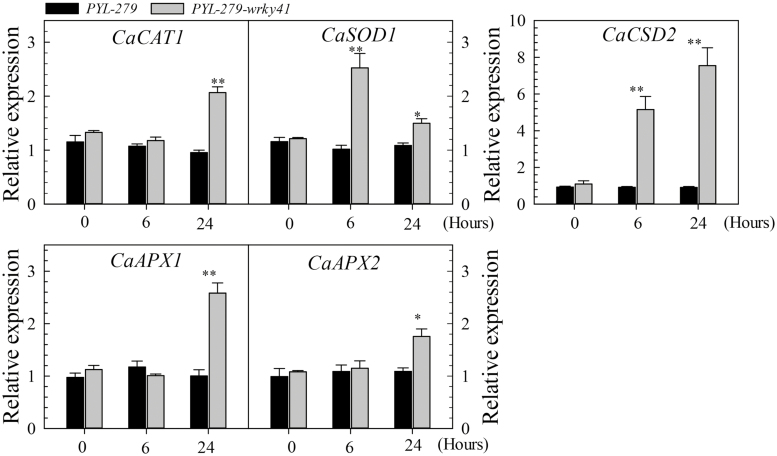
Expression of genes encoding ROS-scavenging enzymes determined by RT–qPCR analysis in *CaWRKY41*-silenced plants 0, 6, and 24 h after treatment with 25 µM CdSO_4_. Data represent the mean ±SE of three biological replicates. Asterisks indicate significant differences compared with control plants (Student’s *t-*test; *P<0.05, **P<0.01).

Upon exposure to Cd stress, *PYL-279-wrky41* plants and detached leaves consistently exhibited attenuated Cd stress-induced chlorosis compared with controls (Fig. 3B–D). *CaWRKY41*-silenced leaves also accumulated less H_2_O_2_ than control leaves under Cd stress (Fig. 3E). Consistently, genes encoding antioxidant enzymes, including CAT (*CaCAT1*), superoxide dismutase (*CaSOD1*), copper zinc superoxide dismutase (*CaCSD2*), and APX (*CaAPX1* and *CaAPX2*), were up-regulated at 24 HPT with Cd stress in the youngest leaves of *PYL-279-wrky41* plants compared with the control. However, no difference in the expression of these genes was detected between the youngest leaves of *PYL-279* and *PYL-279-wrky41* under normal growth conditions (Fig. 4). These results suggest that *CaWRKY41* negatively regulates Cd tolerance, likely by mediating the accumulation of H_2_O_2_ through the transcriptional regulation of antioxidant genes.

### Overexpression of *CaWRKY41* increases sensitivity to Cd in *Arabidopsis* in an H_2_O_2_-dependent manner

**Fig. 5.**
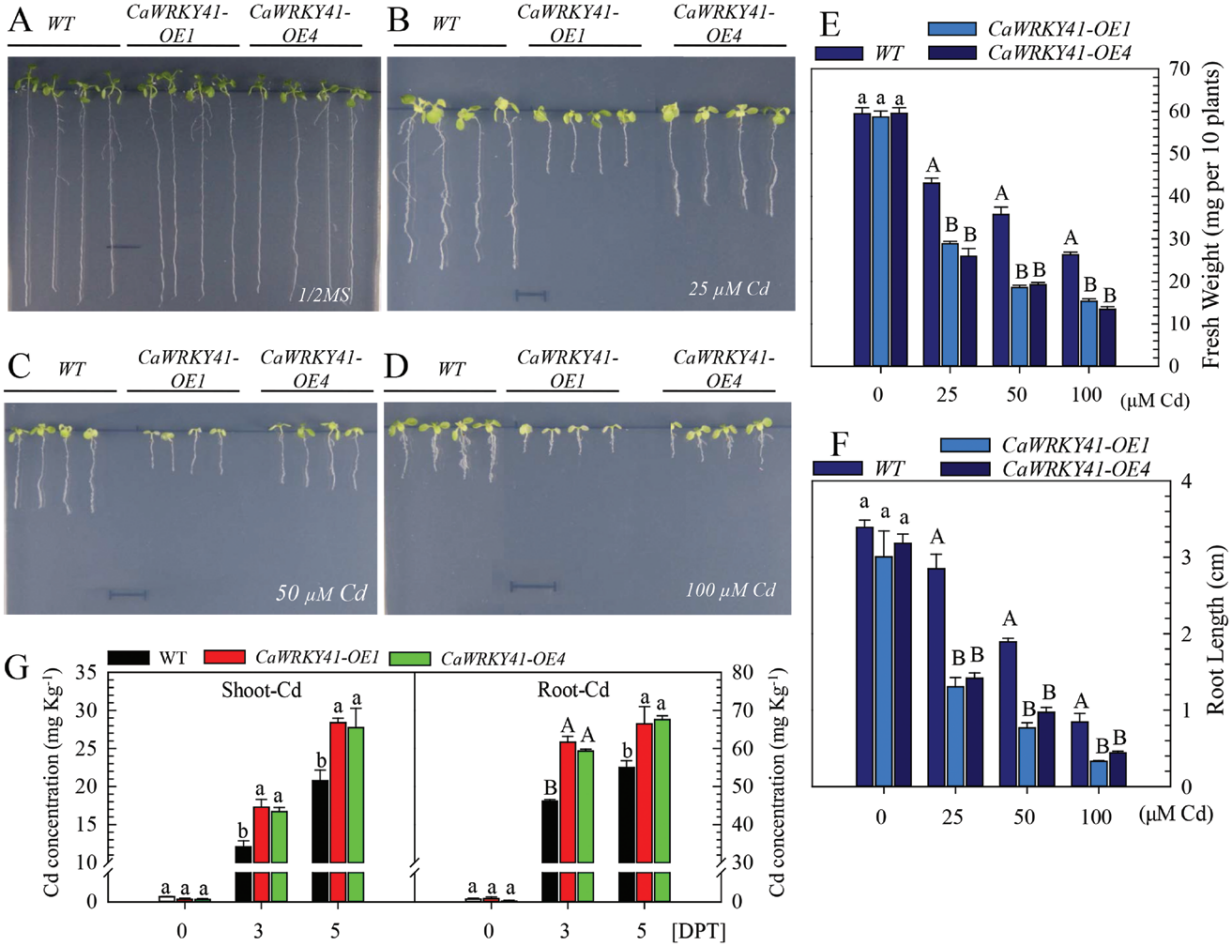
Overexpression of *CaWRKY41* reduces tolerance to Cd stress in transgenic Arabidopsis plants. (A–D) Seedling growth in WT, *CaWRKY41-OE1*, and *CaWRKY41-OE4* lines on ½ MS medium containing (A) 0, (B) 25, (C) 50, and (D) 100 µM CdSO_4_. Representative photographs were taken 8 days after germination. (E) Fresh weight and (F) root length in WT, *CaWRKY41-OE1*, and *CaWRKY41-OE4* plants exposed to Cd stress. (G) Cd concentration in the shoots and roots of WT, *CaWRKY41-OE1*, and *CaWRKY41-OE4* plants after 3 and 5 days of treatment. Data represent the mean ±SE of three biological replicates. Different letters indicate significant differences compared with the control (Tukey’s test; lowercase letters indicate *P*<0.05 and uppercase letters indicate *P*<0.01).

To confirm the results of the *CaWRKY41-*silencing experiments, we performed a gain-of-function analysis by ectopically overexpressing *CaWRKY41* in Arabidopsis. None of the seven *CaWRKY41-*overexpressing T_4_ homozygous transgenic Arabidopsis lines exhibited significant differences in seed germination, seedling growth, or development compared with WT plants under normal conditions (Supplementary Fig. S4C, D), although, as expected, *CaWRKY41-*overexpressing plants exhibited high expression of *CaWRKY41*, as revealed by semi-quantitative PCR (Supplementary Fig. S4E). We randomly selected two independent overexpressing lines (*CaWRKY41-OE1* and *CaWRKY41-OE4*) for further analysis. These *CaWRKY41-OE* lines were more sensitive than the WT to Cd stress (Fig. 5A–D), and had lower fresh weights and shorter roots (Fig. 5E, F).

Next, we compared the growth status of *CaWRKY41-OE1* and *OE4* plants with that of WT plants exposed to excess Cd, or to no Cd, via rapid noninvasive chlorophyll fluorescence imaging. Under normal conditions, there was no marked difference in the fluorescence characteristics of WT and *CaWRKY41-OE* plants (Supplementary Fig.S5A, C, E); however, under Cd stress, *CaWRKY41-OE1* and *OE4* plants exhibited lower chlorophyll fluorescence parameters than WT plants (Supplementary Fig. S5 B, D, F). Furthermore, higher Cd (Fig. 5G) and zinc (Zn) (Supplementary Fig. S6A, B) contents were detected in both the roots and shoots of *CaWRKY41-*overexpressing plants (*OE1* and *OE4*) than in those of the WT after 3 or 5 days of Cd treatment. By contrast, the Fe contents in roots and shoots were similar in *CaWRKY41-OE* and WT plants (Supplementary Fig. S6C, D). Additionally, the *CaWRKY41-OE* lines were more sensitive than the WT plants to excess Zn (Supplementary Fig. S6 E, F).

**Fig. 6.**
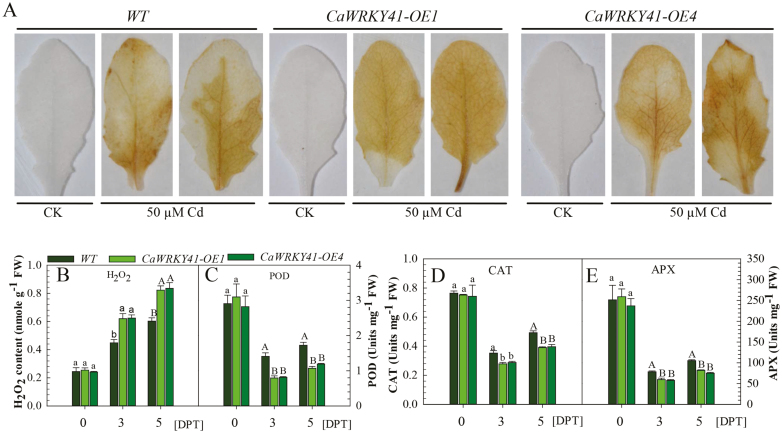
H_2_O_2_ accumulation and ROS-scavenging enzymatic activity in response to Cd stress. (A) H_2_O_2_ production observed via 3, 3′-diaminobenzidine staining in leaves of WT, *CaWRKY41-OE1*, and *CaWRKY41-OE4* plants at 24 h post treatment with 50 µM CdSO_4_. CK, control untreated. (B) Seedling H_2_O_2_ content. DPT, days post treatment. (C) Peroxidase (POD) activity. (D) Catalase (CAT) activity. (E) Ascorbate peroxidase (APX) activity. For B–E, 7-day-old WT, *CaWRKY41-OE1*, and *CaWRKY41-OE4* seedlings were transferred to ½ MS medium without or with 25 µM CdSO_4_ for 3 or 5 days before analysis. Data represent the mean ±SE of three biological replicates. Different letters indicate significant differences compared with the control (Tukey’s test; lowercase letters indicate *P*<0.05 and uppercase letters indicate *P*<0.01).

**Fig. 7.**
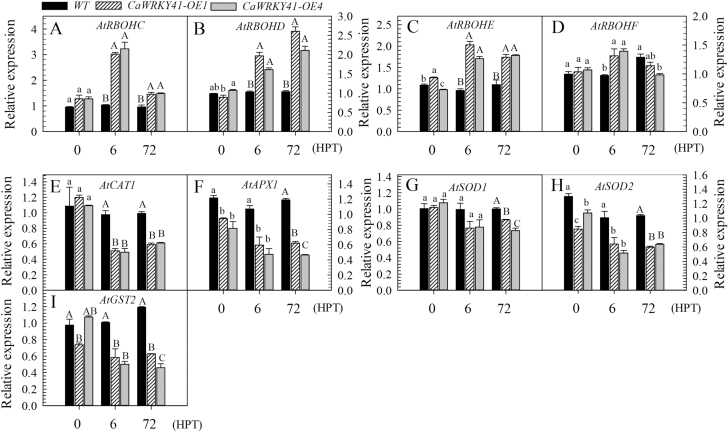
Expression of genes encoding ROS-producing and ROS-scavenging enzymes detected by RT–qPCR analysis in WT, *CaWRKY41-OE1*, and *CaWRKY41-OE4* plants at 0, 6, and 72 h post treatment with Cd. (A–D) Expression of ROS-producing enzyme genes (A) *AtRBOHC*, (B) *AtRBOHD*, (C) *AtRBOHE*, and (D) *AtRBOHF*. (E–I) Expression of ROS-scavenging enzyme genes (E) *AtCAT1*, (F) *AtAPX1*, (F) *AtSOD1*, *AtSOD2*, and (G) *AtGST2.* Data represent the mean ±SE of three biological replicates. Different letters indicate significant differences compared with the control (Tukey’s test; lowercase letters indicate *P*<0.05 and uppercase letters indicate *P*<0.01).

The reduced accumulation of H_2_O_2_ in *CaWRKY41*-silenced leaves compared with control plants under Cd stress suggests that H_2_O_2_ might be involved in *CaWRKY41*-mediated responses to Cd in pepper. To investigate this possibility, we analyzed the effect of *CaWRKY41* overexpression on H_2_O_2_ accumulation in Arabidopsis plants subjected to Cd stress. H_2_O_2_ levels were higher in the leaves of *CaWRKY41*-overexpressing lines (*OE1* and *OE4*) than in those of the WT, as revealed by DAB staining and direct H_2_O_2_ measurements (Fig. 6A, B). Accordingly, the activities of the ROS-scavenging enzymes POD, CAT, and APX were reduced in *OE1* and *OE4* plants compared with WT plants (Fig. 6C–E). By contrast, higher expression of genes associated with ROS production, such as *AtRBOHC* (Macho *et al.*, 2012), *AtRBOHD* (Li *et al.*, 2014; Kadota *et al.*, 2015), *AtRBOHE*, and *AtRBOHF* (Chaouch *et al.*, 2012) (Fig. 7A–D), and lower expression of the ROS-scavenging enzyme genes *AtCAT1*, *AtAPX1*, *AtSOD1*, *AtSOD2*, and *AtGST2* (Fig. 7E–I), were detected in *OE1* and *OE4* plants compared with WT plants at 6 and 72 HPT with Cd. These results suggest that the enhanced accumulation of ROS including H_2_O_2_ in response to *CaWRKY41* overexpression might be due to enhanced ROS production and reduced ROS scavenging, and that elevated H_2_O_2_ levels might contribute to Cd sensitivity in pepper plants.

To test this possibility, we examined whether there was a relationship between H_2_O_2_ accumulation and Cd sensitivity in the *A. thaliana ocp3* (*overexpressor of cationic peroxidase 3*) mutant, which harbors a T-DNA insertion in a homeodomain TF gene involved in increased H_2_O_2_ production in healthy plants (Coego *et al.*, 2005). Mutant *ocp3* plants exhibited shorter primary roots than WT plants under Cd stress (Supplementary Fig. S7), supporting the notion that Cd sensitivity is associated with H_2_O_2_ accumulation. Collectively, these results suggest that the *CaWRKY41-*mediated Cd sensitivity observed in transgenic Arabidopsis is caused by H_2_O_2_ accumulation due to increased H_2_O_2_ production and reduced H_2_O_2_ scavenging.

### Overexpression of *CaWRKY41* increases Cd levels in Arabidopsis by activating Zn transporters

Since we detected higher levels of Cd but not Fe in both the roots and shoots of *CaWRKY41-OE* plants compared with WT upon excess Cd supply, we reasoned that the enhanced Cd sensitivity in response to *CaWRKY41* overexpression might be due to enhanced uptake of Cd. A Cd-specific transporter has not yet been identified, and Cd is thought to be transported by Fe and Zn transporters in plants (Saraswat and Rai, 2011; Barabasz *et al.*, 2016). Therefore, we reasoned that, since Fe levels were not elevated in *CaWRKY41-OE* Arabidopsis plants compared with control plants, Cd might enter *CaWRKY41*-*OE* Arabidopsis plants via Zn transporters.

To test this hypothesis, we measured the expression of genes encoding Zn transporters, including *AtZIP1* (Kawachi *et al.*, 2009), *AtZIP3* (Gustin *et al.*, 2009), *AtZIP4* (Gustin *et al.*, 2009), *AtZIP5* (Gustin *et al.*, 2009), and *AtZIP9* (Gustin *et al.*, 2009) in *CaWRKY41*-*OE* Arabidopsis plants. Only *AtZIP3*, *AtZIP4*, and *AtZIP9* (Supplementary Fig. S8 B, C, E), were up-regulated in these plants compared with controls; the expression of the other genes did not differ from those of controls upon exposure to excess Cd. These results suggest that increased Cd uptake might be due at least in part to the enhanced expression of genes encoding Zn transporters.

### Silencing of *CaWRKY41* confers reduced resistance to *R. solanacearum* inoculation

Our results indicate that H_2_O_2_, which has been implicated in plant immunity (Levine *et al.*, 1994; Alvarez *et al.*, 1998; Torres *et al.*, 2006), is involved in *CaWRKY41*-mediated Cd sensitivity. NADPH oxidases, which contribute to ROS production, have frequently been shown to be involved in plant immunity (Kadota *et al.*, 2015). Thus, we reasoned that *CaWRKY41* might also participate in plant immunity.

**Fig. 8.**
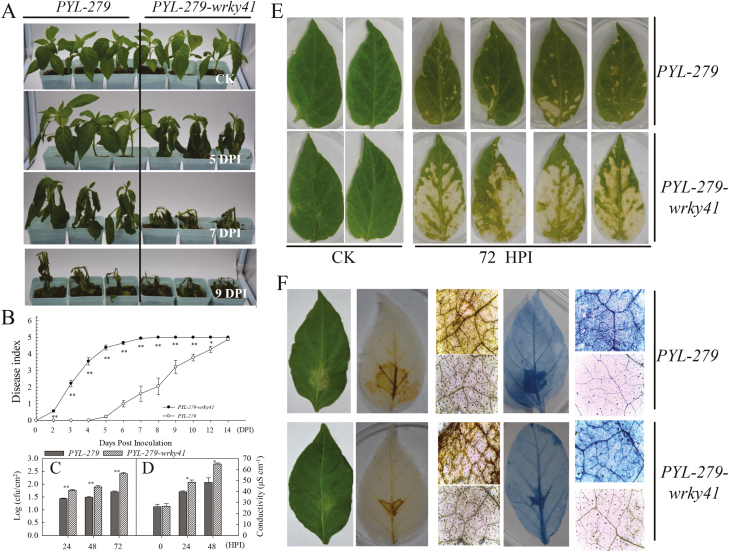
*CaWRKY41* silencing enhances susceptibility to *Ralstonia solanacearum* FJ150501. (A) Appearance of *PYL-279* and *PYL-279-wrky41* pepper plants at 0, 5, 7, and 9 days post inoculation (DPI) with *R. solanacearum*. (B) Disease index scored daily for *R. solanacearum-*inoculated *PYL-279* and *PYL-279-wrky41* pepper plants. (C) Bacterial growth and (D) conductivity (as a measure of electrolyte leakage) in *PYL-279* and *PYL-279-wrky41* pepper leaves following *R. solanacearum* inoculation. HPI, hours post inoculation. Data represent the mean ±SE of three biological replicates. Asterisks indicate significant differences compared with control plants (Student’s *t-*test; *P<0.05, **P<0.01). (E) Effect of *R. solanacearum* on leaves isolated from *PYL-279* and *PYL-279-wrky41* plants. *R. solanacearum* was collected from stem exudates or the vascular portions of infected pepper leaves, and the appearance of symptoms was observed 72 HPI. CK, control untreated. (F) Decreased H_2_O_2_ levels and cell death in the leaves of *PYL-279-wrky41* pepper plants compared with *PYL-279* 24 h after inoculation with *R. solanacearum*.

To test this possibility, we monitored changes in *CaWRKY41* expression in response to *R. solanacearum* inoculation. *CaWRKY41* was strongly induced by *R. solanacearum* inoculation, with peak expression detected at 6 h post inoculation (Supplementary Fig. S9A). In addition, analysis of *CaWRKY41*-silenced pepper plants, in which *CaWRKY41* expression was approximately 3.2- and 3.3-fold lower than the control (*PYL-279*) under pathogen inoculation and non-inoculation conditions, respectively (Supplementary Fig. S9B), showed that *CaWRKY41* silencing increased susceptibility to *R. solanacearum* compared with *PYL-279* plants at 5, 7, and 9 days post inoculation (Fig. 8A). Consistently, *PYL-279-wrky41* plants had a higher disease index, higher rate of *R. solanacearum* growth, and higher level of electrolyte leakage compared with *PYL-279* plants (Fig. 8B–D). In addition, more serious symptoms of bacterial wilt were observed in the detached youngest leaves of *PYL-279-wrky41* compared with *PYL-279* plants after infiltration of an *R. solanacearum* suspension for at least 30 min, while no difference was observed in untreated leaves (Fig. 8E).

DAB staining revealed *R. solanacearum*-triggered H_2_O_2_ production in *PYL-279* plants, but much less H_2_O_2_ accumulation was detected in *R. solanacearum*-inoculated *CaWRKY41*-silenced pepper leaves than in *PYL-279* leaves. Similarly, much higher levels of cell death (as revealed by Trypan blue staining) were triggered by *R. solanacearum* inoculation in the youngest leaves of *PYL-279* plants at 24 h post inoculation (Fig. 8F) compared with *PYL-279-wrky41*. These results indicate that the role of *CaWRKY41* as a positive regulator of plant immunity is also associated with H_2_O_2_ signaling.

### The response of pepper to Cd stress is closely associated with the response to *R. solanacearum* inoculation

Our data show that overexpression of *CaWRKY41* increases sensitivity to Cd in Arabidopsis in an H_2_O_2_-dependent manner, and that silencing of *CaWRKY41* enhances susceptibility to *R. solanacearum* infection and reduces H_2_O_2_ accumulation. Specifically, we found that *AtOCP3,* an important modulator of plant immunity that encodes a protein that catalyzes H_2_O_2_ production (Coego *et al.*, 2005; Ramirez *et al.*, 2010; Garcia-Andrade *et al.*, 2011), also confers Cd sensitivity. We reasoned that H_2_O_2_ might act as a crucial signaling component that coordinates the response to Cd stress and *R. solanacearum* inoculation in pepper and, if so, that these responses are closely related.

To test this possibility, we monitored the growth of *R. solanacearum* in the leaves of pepper plants under Cd stress, and found that the growth of the pathogen was significantly repressed by Cd stress (Supplementary Fig. S9C). Furthermore, expression of *CaPR1*, *CaPR4*, and *CaNPR1* was induced under Cd toxicity in pepper plants (Supplementary Fig. S9 D–F). On the other hand, when pepper plants were challenged with *R. solanacearum*, the Cd contents in the roots and leaves of *R. solanacearum*-inoculated pepper plants were significantly higher than those of mock-treated control plants (Supplementary Fig. S9G, H). Together, these data indicate that the responses of pepper to Cd stress and *R. solanacearum* inoculation are closely related.

## Discussion

Although plant immunity and Cd tolerance have been intensively studied in the past few decades, and several proteins have been implicated in both of these processes (Mirouze *et al.*, 2006; Kim *et al.*, 2007; Kuhnlenz *et al.*, 2015; Campe *et al.*, 2016; Peris-Peris *et al.*, 2017), little is known about the connections between the two processes. In the present study, we provide evidence that both immunity and Cd uptake in pepper are coordinately regulated by *CaWRKY41* and are dependent on the ROS signaling pathway.

### Responses of pepper to *R. solanacearum* inoculation and Cd are coordinately regulated by *CaWRKY41*

We analyzed the expression of eight group III *WRKY* genes in the roots and leaves of pepper plants grown in the presence of excess Cd or under Fe deficiency, since the response of plants to Fe deficiency was previously shown to be related to the response to excess Cd (Nakanishi *et al.*, 2006; Han *et al.*, 2014; Mendoza-Cozatl *et al.*, 2014). Among these eight genes, only *CaWRKY41* was up-regulated in roots and leaves by both excess Cd exposure and Fe deficiency (Fig. 1). In addition, *CaWRKY41* was induced by *R. solanacearum* inoculation (Supplementary Fig. S9A), pointing to a role for *CaWRKY41* in the crosstalk between the response to excess Cd exposure and *R. solanacearum* inoculation in pepper. Gain- and loss-of-function analyses confirmed this speculation: *CaWRKY41*-silenced pepper plants showed substantially enhanced sensitivity to *R. solanacearum* inoculation (Fig. 8A), as also revealed by lighter Trypan blue staining compared with *PYL*-*279* plants when challenged with *R. solanacearum* (Fig. 8F). In addition, the growth rate of *R. solanacearum* and the disease index (indicative of the severity of symptoms of infection) was higher in *CaWRKY41*-silenced pepper plants than in *PYL*-*279* plants (Fig. 8B, C). Moreover, the leaves of *CaWRKY41*-silenced pepper plants showed enhanced tolerance to Cd (Fig. 3B–D), while *CaWRKY41*-overexpressing *Arabidopsis* plants exhibited enhanced sensitivity to Cd (Fig. 5A–D), with these plants having a lower fresh weight and shorter primary root than WT plants (Fig. 5E, F).

Together, our findings indicate that *CaWRKY41* is a positive regulator of immunity and a negative regulator of Cd tolerance in pepper. Crosstalk between biotic and abiotic stress responses is thought to be involved in coordinately regulating plant responses to multiple environmental stresses (Fujita *et al.*, 2006; Wu *et al.*, 2009). Although the synergistic effect of Cd and *Botrytis* infection on *PDF1.2* expression (Cabot *et al.*, 2013) and the differential regulation of Cd uptake in response to SA application in plants (Kovacik *et al.*, 2009) have been previously reported, little is known about the crosstalk between Cd toxicity and pathogen responses. Furthermore, members of the WRKY TF family have been implicated in plant immunity, but only a few WRKY TFs, such as *T. hispida* WRKY7 (Yang *et al.*, 2016) and *Z. mays* WRKY4 (Hong *et al.*, 2017), have been shown to positively regulate plant tolerance to Cd toxicity. The results of the current study strongly suggest that *CaWRKY41* plays a role in the crosstalk between the response of pepper to *R. solanacearum* infection and excess Cd exposure.

### *R. solanacearum* inoculation and excess Cd activate a positive feedback loop between *CaWRKY41* expression and H_2_O_2_ accumulation

Although bursts of ROS including H_2_O_2_ have been shown to be involved in plant responses to pathogen attack (Torres *et al.*, 2006; Vellosillo *et al.*, 2010) and exposure to Cd toxicity (Garnier *et al.*, 2006; Heyno *et al.*, 2008), and the role of H_2_O_2_ as a signaling molecule in plant immunity is well established (Alvarez *et al.*, 1998; Qi *et al.*, 2017), the role of H_2_O_2_ in plant responses to Cd toxicity has remained elusive.

The results of the current study indicate that both exposure to excess Cd and *R. solanacearum* inoculation trigger H_2_O_2_ accumulation in pepper plants. The enhanced H_2_O_2_ accumulation might induce the expression of *CaWRKY41*, as exogenous application of H_2_O_2_ significantly increases *CaWRKY41* expression (Fig. 2D), which in turn triggers H_2_O_2_ accumulation in Arabidopsis under Cd stress, as revealed by DAB staining and direct H_2_O_2_ measurements (Fig. 6A, B). These results suggest that there is a positive feedback loop between *CaWRKY41* expression and H_2_O_2_ accumulation during the response to *R. solanacearum* inoculation and excess Cd exposure in pepper. Similar positive feedback loops are common in plant responses to pathogens or other abiotic stresses and are believed to be crucial for amplifying defense signaling (Wang *et al.*, 2014; Cai *et al.*, 2015; Shen *et al.*, 2016; Guo *et al.*, 2017; Ren *et al.*, 2018). In plants, H_2_O_2_ is a general signaling molecule in the response to pathogen or abiotic stresses and is coupled with large-scale transcriptional reprogramming (Yang *et al.*, 2013). However, it is unclear how H_2_O_2_ signaling is linked to specific TFs. It was recently reported that oxidation of the BRASSINAZOLE-RESISTANT1 (BZR1) transcription factor can be induced by H_2_O_2_, and that this plays a major role in regulating gene expression (Tian *et al.*, 2018).

Further research is required to elucidate the mechanism underlying H_2_O_2_-mediated transcriptional modulation of *CaWRKY41* expression during the response to Cd stress and *R. solanacearum* infection in pepper. H_2_O_2_ accumulation was attributed to its enhanced production and reduced degradation due to the enhanced expression of *CaWRKY41*, since the genes encoding NADPH oxidases (associated with ROS production), including *AtRBOHC* (Macho *et al.*, 2012), *AtRBOHD* (Li *et al.*, 2014; Kadota *et al.*, 2015), *AtRBOHE*, and *AtRBOHF* (Chaouch *et al.*, 2012) were up-regulated in Arabidopsis plants overexpressing *CaWRKY41* (Fig. 7A–D). These results are consistent with the finding that NADPH oxidases differentially regulate ROS production and are significantly up-regulated by Cd exposure (Gupta *et al.*, 2017). Furthermore, H_2_O_2_ accumulation has been found to be dependent on or closely correlated to NADPH oxidase (Foreman *et al.*, 2003). By contrast, genes encoding antioxidant enzymes, including *POD*, *CAT*, and *APX* (Smeets *et al.*, 2013), were significantly down-regulated in response to *CaWRKY41* overexpression in Arabidopsis (Fig. 6C–E, Fig. 7E–I). Similarly, it was reported that repression of *CATALASE2* (*CAT2*) resulted in H_2_O_2_ accumulation, and that inhibition of H_2_O_2_ degradation conferred enhanced disease resistance (Yuan *et al.*, 2017).

We speculate that exposure to excess Cd triggers H_2_O_2_ accumulation, and that H_2_O_2_, and therefore the expression of *CaWRKY41*, might confer Cd sensitivity and resistance to *R. solanacearum*. In support of this notion, the Arabidopsis *ocp3* mutant, which produces high levels of H_2_O_2_ and exhibits increased resistance to the necrotrophic pathogens *Botrytis cinerea* and *Plectosphaerella cucumerina* (Coego *et al.*, 2005), exhibited enhanced sensitivity to excess Cd compared with control plants in the present study (Supplementary Fig. S7). In addition, Cd exposure repressed the growth of *R. solanacearum* in inoculated pepper plants (Supplementary Fig. S9C). By contrast, *R. solanacearum* inoculation increased Cd uptake by the roots and leaves of pepper plants exposed to excess Cd (Supplementary Fig. S9G, H). Together, these results strongly suggest that H_2_O_2_ accumulation increases plant immunity and plant sensitivity to excess Cd.

### *CaWRKY41* likely mediates Cd sensitivity by enhancing Cd uptake via enhanced Zn transporter activity

Increased Cd uptake or reduced levels of Cd detoxification result in cellular damage in plants (Schutzendubel *et al.*, 2001). We found that Cd levels in both the roots and shoots of *CaWRKY41*-overexpressing Arabidopsis plants were significantly higher than those of WT plants (Fig. 5G), indicating that the susceptibility of *CaWRKY41*-overexpressing Arabidopsis plants to Cd stress is due to their high Cd contents.

Our findings suggest that the enhanced Cd contents might be due to the up-regulation of various Zn transporter genes, such as *AtZIP3*, *AtZIP4*, and *AtZIP9*, by CaWRKY41 (Supplementary Fig. S8B, C, E). Indeed, uptake of Cd by Zn and Fe transporters has previously been suggested (Saraswat and Rai, 2011; Barabasz *et al.*, 2016), and Fe content was found to increase in Arabidopsis roots and to vary in accordance with the period and concentration of Cd treatment (Gupta *et al.*, 2017). However, although *CaWRKY41* was activated by Fe deficiency, the Fe content of *CaWRKY41*-overexpressing Arabidopsis plants did not significantly differ from that of control plants (Supplementary Fig. S6C, D). It is puzzling from an evolutionary point of view why *CaWRKY41* would positively regulate disease resistance in pepper plants but promote the absorption of Cd and enhance sensitivity to Cd, which might reduce the adaptability of the plant to a heavy-metal-contaminated environment. We speculate that *CaWRKY41* might have evolved to coordinate plant immunity and the absorption of essential ions, including Zn, by modulating the activity of specific ion transporters. Indeed, Zn is required for the functioning of Zn binding motif-containing proteins associated with disease resistance, including WRKY TFs (Duan *et al.*, 2007), Rar1 (Shirasu *et al.*, 1999; Muskett *et al.*, 2002; Wang *et al.*, 2017), and R proteins (Yang *et al.*, 2010; Bi *et al.*, 2011), which play important roles in plant immunity. However, some of these ion transporters can be hijacked by Cd, which has only recently been released into the environment as a result of modern industrial practices, suggesting that plants have not yet evolved a counterstrategy to distinguish between Zn and Cd.

**Fig. 9.**
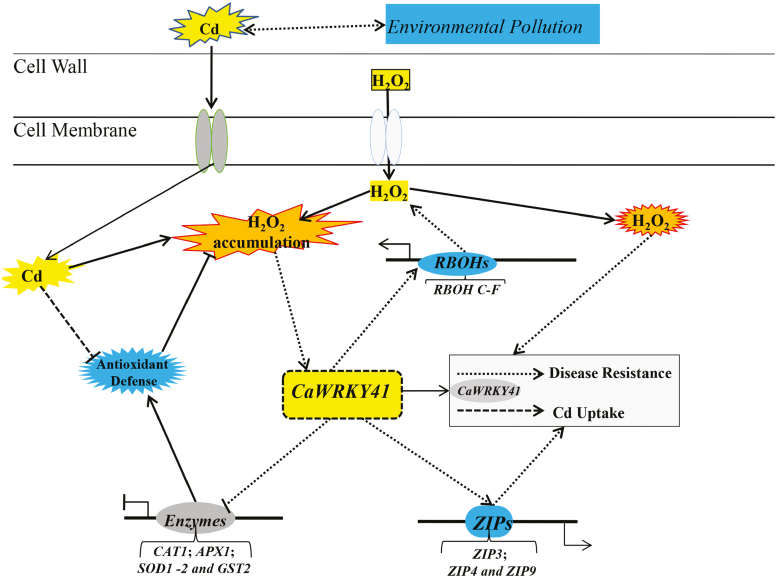
Working model for the role of *CaWRKY41* in regulating Cd sensitivity and *R. solanacearum* resistance in pepper. Cd toxicity induces H_2_O_2_ production and inhibits the activity of ROS-scavenging enzymes, leading to accumulation of H_2_O_2_ and up-regulation of *CaWRKY41*. Subsequently, CaWRKY41 directly or indirectly activates the expression of ROS-producing genes (*RBOH C-F*) and Zn transporters (*ZIP3*, *ZIP5*, and *ZIP9*), and inhibits the expression of ROS-scavenging enzymes (*CAT1*, *APX1*, *SOD1*, *SOD2*, and *GST2*). Finally, a positive feedback loop between H_2_O_2_ accumulation and *CaWRKY41* up-regulation coordinates the responses of pepper to *R. solanacearum* infection and Cd toxicity.

Based on these findings, we propose a working model (Fig. 9) in which H_2_O_2_ accumulation and the expression of *CaWRKY41*, as well as a positive feedback loop between these processes, are induced by *R. solanacearum* infection or excess Cd exposure. The increase in H_2_O_2_ accumulation and *CaWRKY41* expression enhance plant immunity and sensitivity to excess Cd exposure by increasing Cd uptake via Zn transporters.

## Supplementary data

Supplementary data are available at *JXB* online.

Fig. S1. Phylogenetic analysis of eight pepper group III WRKY proteins and Arabidopsis and tomato group III WRKY proteins.

Fig. S2. Cd stress and Fe deficiency promotes H_2_O_2_ accumulation.

Fig. S3. GUS expression in transgenic *pCaWRKY41::GUS* Arabidopsis plants under normal growth conditions.

Fig. S4. CaWRKY41 is a transcriptional activator localized to the nucleus.

Fig. S5. Analysis of the effects of Cd stress on plant growth using chlorophyll fluorescence imaging before the appearance of visible effects on plant growth.

Fig. S6. Effect of Cd treatment on Zn concentrations in Arabidopsis.

Fig. S7. The Arabidopsis *ocp3* mutant shows reduced tolerance to Cd stress.

Fig. S8. RT–qPCR analysis of the *ZIP* members involved in Zn uptake.

Fig. S9. Cd inhibits *R. solanacearum* growth and *R. solanacearum* infection increases Cd uptake.

Table S1. Sequences of primers used in this study.

Table S2. *CaWRKY* group III genes.

Table S3. Analysis of the *C/S*-elements in the 2 kb promoter fragment of *CaWRKY* group III genes.

Supplementary Figures S1-S9

Supplementary Tables S1-S3

## Abbreviations:

ROS: reactive oxygen species
TF: Transcription factor
VIGS: virus-induced gene silencing
