# Supplementary material for: A feedback loop between CaWRKY41 and H2O2 coordinates the response to Ralstonia solanacearum and excess cadmium in pepper
Source: J Exp Bot. 2019 Jan 14;70(5):1581–95. doi: 10.1093/jxb/erz006 (PMC6416791; doi:10.1093/jxb/erz006)
Supplement: Supplementary Figures S1-S9 [file erz006_suppl_supplementary_figures_s1-s9.pdf]

Supplementary data

Table S1. Sequences of primers used in this study.

Table S2. *CaWRKY* groupIII genes.

Table S3. Analysis of the *C/S*-elements in the 2-kb promoter fragment of *CaWRKY* groupIII genes.

Table S1. Sequences of primers used in this study

Primer Name	Forward (5′-3′)	Reverse (5′-3′)
**For cloning***		
CaWRKY41 (CDS)	GGGGACAAGTTTGTACAAAAAAGCAGGCTTCATGGAGAAAGTTAAAGGATTGG	GGGGACCACTTTGTACAAGAAAGCTGGGTCTTAACTAAAGAACTCTTCAATG
CaWRKY41 (promoter)	GGGGACAAGTTTGTACAAAAAAGCAGGCTGAATAGAACCCTATCCGACG	GGGGACCACTTTGTACAAGAAAGCTGGGTTTTGTTGTAAAAACTCAAAT
CaWRKY41 (VIGS)	GGGGACAAGTTTGTACAAAAAAGCAGGCTTCCAAAAACGCCAGAAACACA	GGGGACCACTTTGTACAAGAAAGCTGGGTCAATCCCAATCTCCGACAAATGA
**For RT-qPCR**		
CaWRKY41	CACCAAACCATGACGCAGAA	TGAGCAGTGCAGAGTCATGT
CaWRKY53a	TGGAGAAAGTACGGCCAGAA	TCTCGTTTCTCTGGCGATGT
CaWRKY52	TCGTGGAAGCTTCAAAGTCA	ACACATGAAACACACTACCGA
CaWRKY53	CTCAACAGTCCACTCCTCGA	TCTCCGCACTGACTTTGACT
CaWRKY54	TGTCTCTCCTACAACCCCTG	TCTAGCTCCACACGTTCAAGT
CaWRKY70	TGGAGGAAATACGGGCAGAA	TGCTTCCGAATCTTTGCTGG
CaWRKY30	GGTCATCACTCGTGCACAAC	CATCGGATTGACTTTGGCCA
CaWRKY41a	TCCCATACTTGTCGCGAAGT	GGCTGCCGGAAACTGATAAG
CaCAT1	TGTTGCTGGTGTTGGTGTTGGT	GCCTCTCCTAGACGGCCTTTCA
CaAPX1	CAGGCAGAGGCAAGGGAGGTAA	CCCTACGAGCGAGCCTACGAAT
CaAPX2	TGATGCTCTGCCAATATCGGAT	CCAATTCCTCCCATGCCAAC
CaCSD2	TGTGGATGTGGCTTTGGTTCCC	TGAGGAAGGGAGTTTGCGACAC
CaSOD1	ACACAACTCCACCATAGAGCCT	CCACCAACAATCGCACCAACAT
CaPR1	GCCGTGAAGATGTGGGTCAATGA	TGAGTTACGCCAGACTACCTGAGTA
CaPR4	CAACCCGCAGAACATCAACTGG	CCTCAAGCATCTACCGCAAGCA
CaNPR1	ACTTCTTCGCCGACGCCAAG	GCCAACACATTCACCAGAGCATC
CaActin	AGGGATGGGTCAAAAGGATGC	GAGACAACACCGCCTGAATAGC
AtRBOHC	TCACCAGAGACTGGCACAATAAA	GATGCTCGACCTGAATGCTC
AtRBOHD	TATGCATCGGAGAGGCTGCT	TAGAGACAACACGTTCCCGGG
AtRBOHE	GTGATGCAAGATCAACCCTGA	GCCTTGCAAAATGTGTTCTCA
AtRBOHF	GGTGTCATGAACGAAGTTGCA	AATGAGAGCAGAACGAGCATCA
AtCAT1	AAGTGCTTCATCGGGAAGGA	CTTCAACAAAACGCTTCACGA
AtAPX1	TGCCACAAGGATAGGTCTGG	CCTTCCTTCTCTCCGCTCAA

AtSOD1	TCCATGCAGACCCTGATGAC	CCTGGAGACCAATGATGCC
AtSOD2	GAGCCTTTGTGGTTCACGAG	CACACCACATGCCAATCTCC
AtGST2	ATCACCAGTTCGACCCAGTG	CTCCTCTTCTGCAACAACGG
AtZIP1	GGACACACACATGGTTCGAC	GATAGTGCAGCCATGAGTGG
AtZIP3	CAGAAACATGTTTCTTCTTCGTCAC	CGCAATAAATCCGGTGAACG
AtZIP4	GATCTTCGTCGATGTTCTTTGG	TGAGAGGTATGGCTACACCAGCAGC
AtZIP5	CGGGATTGTTGGCGTGGAAT	CCAAGACCCTCGAAGCATTG
AtZIP9	CAATAATCATAGGAATATCGCTTGG	AGAAAGCCATCATGGCAGAT
AtUBQ10	GGCCTTGTATAATCCCTGATGAATA	AAAGAGATAACAGGAACGGAAACATA

*Sequences of the recombination sites of the Gateway system are underlined

Table S2. *CaWRKY* groupIII genes

Gene symbol	Gene locus	Domain in homologous gene in *Arabidopsis thaliana*	Identity	Protein encoded by homologous gene in *Arabidopsis thaliana*	Identity	Domain in homologous gene in *Solanum lycopersicum*	Identity	Protein encoded by homologous gene in *Solanum lycopersicum*	Identity	WRKY domain		Location	Group
										Conserved heptapeptide	Zinc-finger type		
CaWRKY54	CA01g01280	AtWRKY41&53	77.42%	AtWRKY53	27.38%	SlWRKY54	93.65%	SlWRKY54	76.33%	WRKYGQK	C_2HC	2038036-2040409	III
CaWRKY53a	CA01g34460	AtWRKY53	77.42%	AtWRKY53	30.71%	SlWRKY53	85.71%	SlWRKY53	57.22%	WRKYGQK	C_2HC	272190035-272192286	III
CaWRKY53	CA01g34470	AtWRKY53	80.33%	AtWRKY53	29.29%	SlWRKY53	90.48%	SlWRKY53	81.32%	WRKYGQK	C_2HC	272193930-272196370	III
CaWRKY41a	CA01g34480	AtWRKY41	62.90%	AtWRKY41	21.92%	SlWRKY54	71.43%	SlWRKY53	45.73%	WRKYGQK	C_2HC	272199809-272204804	III
CaWRKY70	CA03g12230	AtWRKY70	60.32%	AtWRKY70	18.53%	SlWRKY80	92.06%	SlWRKY80	64.04%	WRKYGQK	C_2HC	92810679-92814562	III
CaWRKY52	CA03g19220	AtWRKY41	82.26%	AtWRKY41	25%	SlWRKY52	93.65%	SlWRKY52	75.20%	WRKYGQK	C_2HC	211827372-211829150	III
CaWRKY41	CA08g08240	AtWRKY41&53	74.19%	AtWRKY41&53	21.43%	SlWRKY41	90.48%	SlWRKY41	75.66%	WRKYGQK	C_2HC	125184240-125186984	III
CaWRKY30	CA10g06890	AtWRKY30&64	59.68%	AtWRKY70	13.55%	SlWRKY81	68.84%	SlWRKY81	30.17%	WRKYGQK	C_2HC	74808336-74809350	III

Table S3. Analysis of the *C/S*-elements in the 2-kb promoter fragment of *CaWRKY* groupIII genes

Gene symbol	Gene locus	ABRE	ARE	AuxRR-core	CGTCA-motif	ERE	GARE-motif	HSE	LTR	MBS	TC-rich repeats	TCA -element	TGA -element	W-box	P-box	Total
CaWRKY54	*CA01g01280*	0	0	0	1	0	0	3	0	1	3	2	0	0	0	10
CaWRKY53	*CA01g34460*	0	2	0	2	1	1	0	0	3	1	0	0	1	0	11
CaWRKY53a	*CA01g34470*	0	1	0	0	0	0	2	0	0	2	0	0	0	0	5
CaWRKY41a	*CA01g34480*	1	0	1	4	0	0	0	1	1	0	1	0	1	2	12
CaWRKY70	*CA03g12230*	0	2	0	4	0	0	0	0	2	2	2	1	1	1	15
CaWRKY52	*CA03g19220*	0	0	0	0	2	0	2	0	2	2	1	1	1	0	11
CaWRKY41	*CA08g08240*	2	0	0	0	0	0	0	1	2	4	1	1	3	0	14
CaWRKY30	*CA10g06890*	0	0	0	0	0	0	3	0	4	0	1	1	1	0	10
