# Supplementary material for: A feedback loop between CaWRKY41 and H2O2 coordinates the response to Ralstonia solanacearum and excess cadmium in pepper
Source: J Exp Bot. 2019 Jan 14;70(5):1581–95. doi: 10.1093/jxb/erz006 (PMC6416791; doi:10.1093/jxb/erz006)
Supplement: Supplementary Tables S1-S3 [file erz006_suppl_supplementary_tables_s1-s3.pdf]

Supplementary data

Figure S1. Phylogenetic analysis of eight pepper groupIII WRKY proteins and *Arabidopsis* and tomato groupIII WRKY proteins. The trees were constructed using MEGA6.06.

A, Phylogenetic tree for groupIII WRKY proteins from pepper, tomato, and *Arabidopsis*.

B, Phylogenetic tree for groupIII WRKY domains from pepper, tomato, and *Arabidopsis*. Amino acid sequences labeled with red diamonds, yellow circles, and green triangles represent WRKYs from pepper, tomato, and *Arabidopsis*, respectively.

Figure S2. Cd stress and Fe deficiency promotes H_2O_2 accumulation.

A and B, H_2O_2 levels analyzed by DAB staining (brown) at the indicated time points after treatment with 25 μM $CdSO_4$ and Fe deficient (−Fe) nutrient solution, respectively.

C & D, Leaf yellowing phenotype observed in newly emerging leaves of pepper plants at the eight-leaf stage after Fe deficiency treatment.

E, Excess Cd-triggered H_2O_2 accumulation was inhibited by treatment with 10 mM ascorbic acid (AsA).

Figure S3. GUS expression in transgenic *pCaWRKY41::GUS Arabidopsis* plants under normal growth conditions.

A to F, Photographs of seedlings at 1(A), 3(B), 4 (C), 5 (D), and 6 (E) days after germination (DAG). F, Cleared shoot corresponding to E.

G, Mature leaves and petiole, which may also include vascular bundles.

H and I, Flowers.

J, Siliques.

K, Amino acid sequence analysis of CaWRKY41. A nuclear localization signal and WRKY domain were identified in CaWRKY41; the amino acid sequence is shown in bold and underlined in green. The domain underlined in black contains a highly conserved heptapeptide stretch (WRKYGQY) at its N-terminus, followed by a zinc-finger motif (Cx7Cx23HxC) and C-C-H-C, as indicated by black triangles.

Figure S4. CaWRKY41 is a transcriptional activator localized to the nucleus.

A, Subcellular localization of CaWRKY41-GFP and GFP transiently expressed under the control of the *35S* promoter in *N. benthamiana* epidermal cells. DAPI, 4,6-diamidino-2-phenylindole (nucleus stain).

B, Transcriptional activation assay of CaWRKY41 in yeast cells. LacZ reporter gene expression is indicated by blue staining.

C, Growth of 5-week-old WT and *CaWRKY41*-overexpression lines.

D, Leaves isolated from the plants shown in C.

E, Semi-quantitative PCR analysis of *CaWRKY41* expression in eight WT and *CaWRKY41*-overexpression lines.

Figure S5. Analysis of the effects of Cd stress on plant growth using chlorophyll fluorescence imaging before the appearance of visual effects on plant growth.

A & B, Ten-day-old WT, *CaWRKY41-OE1*, and *CaWRKY41-OE4 Arabidopsis* plants grown on ½ MS medium without or with 60 μM $CdSO_4$. Images of the chlorophyll fluorescence parameter values, *Fv/Fm*, for the plants shown in A and B are shown in C and D, respectively. The data in the images of *Fv/Fm* shown in A and B were mapped to the color palette histograms in E and F, respectively.

Figure S6. Effect of Cd treatment on Zn concentrations in *Arabidopsis*.

A to D, Zn and Fe concentrations in shoots and roots of WT, *CaWRKY41-OE1*, and *CaWRKY41-OE4* plants after 3 and 5 days of treatment with Cd stress. E & F, Seedling growth of WT, *CaWRKY41-OE1*, and *CaWRKY41-OE4 Arabidopsis* plants on ½ MS medium containing 0 and 200 μm $ZnSO_4$. The data represent the mean ± SE of three biological replicates. Different letters indicate significant differences compared to the control (Tukey's test; lowercase letters indicate $P < 0.05$ and uppercase letters indicate $P < 0.01$).

Figure S7. The *Arabidopsis ocp3* (*overexpressor of cationic peroxidase 3*) mutant shows reduced tolerance to Cd stress.

A, B, and C, Seedling growth of WT and *opc3* on ½ MS medium containing 0, 30, and 60 μM

$CdSO_4$. Representative photographs were taken 8 days after germination (DAG).

D, Root elongation in WT and *opc3* in response to Cd stress. Data represent the mean ± SE of three biological replicates, and asterisks indicate a significant difference compared with WT (Student's *t*-test; *$P < 0.05$ or **$P < 0.01$).

Figure S8. RT-qPCR analysis of the *ZIP* members involved in Zn uptake.

A to E, Expression of the Zn transporter genes *AtZIP1*, *AtZIP3*, *AtZIP4*, *AtZIP5*, and *AtZIP9*, detected by RT-qPCR analysis in WT, *CaWRKY41-OE1*, and *CaWRKY41-OE4* plants at 0, 6, and 72 hours post treatment with Cd stress. The data represent the mean ± SE of three biological replicates. Different letters indicate significant differences compared to the control (Tukey's test; lowercase letters indicate $P< 0.05$ and uppercase letters indicate $P< 0.01$).

Figure S9. Cd inhibits *Ralstonia solanacearum* growth and *R. solanacearum* infection increases Cd uptake.

A, *CaWRKY41* expression detected by RT-qPCR analysis in pepper plants at the indicated time points post inoculation with *R. solanacearum* FJ150501.

B, *CaWRKY41* expression in *PYL-279* and *PYL-279-wrky41* pepper leaves at the indicated time points post inoculation with *R. solanacearum*. Relative expression of *CaWRKY41* in *PYL-279-wrky41* plants was compared to that of control (*PYL-279*) plants, which was set to 1.

C, *R. solanacearum* growth at 24 or 48 hours post treatment with Cd stress.

D to F, Expression of the defense marker genes *CaPR1*, *CaPR4*, and *CaNPR1*, detected by RT-qPCR analysis in pepper plants at 0, 24, and 48 hours post treatment with 10 or 50 μM Cd. The data represent the mean ± SE of three biological replicates, and asterisks indicate a significant difference compared to the control (Student's *t*-test; *$P < 0.05$ or **$P < 0.01$).

G & H, Cd concentration in the roots and leaves of pepper plants at the indicated time points post inoculation with *R. solanacearum*, respectively. The data represent the mean ± SE of three biological replicates. Different letters indicate significant differences compared to the control (Tukey's test; lowercase letters indicate $P< 0.05$ and uppercase letters indicate $P< 0.01$).

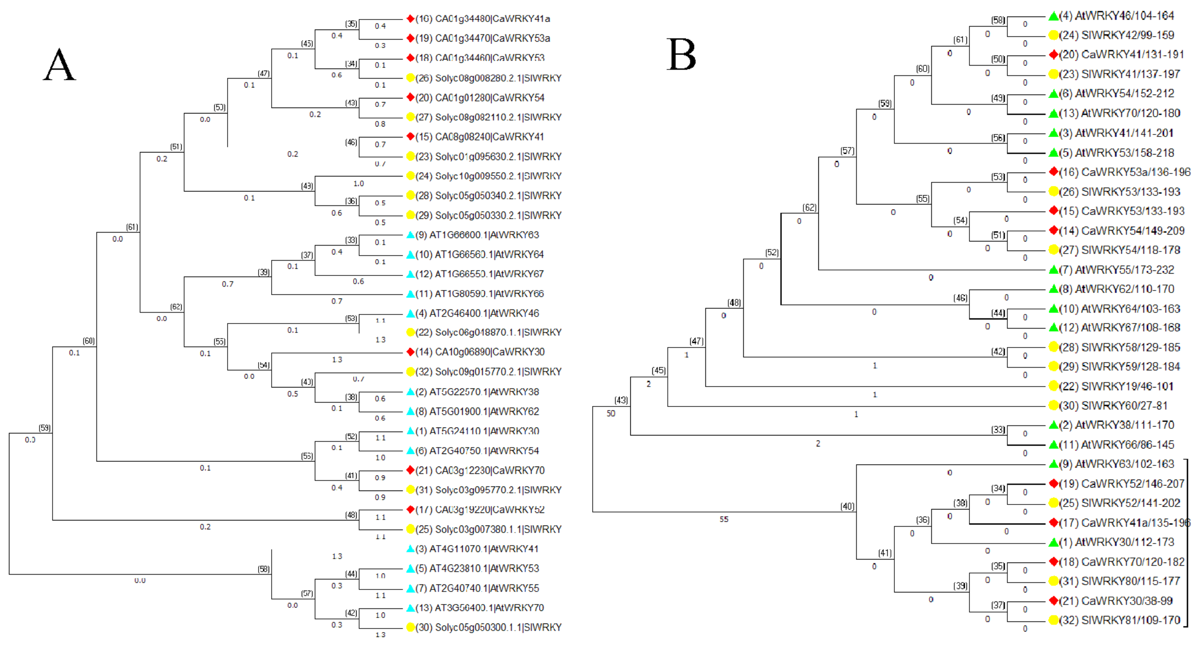

Figure S1. Phylogenetic analysis of eight pepper groupIII WRKY proteins and *Arabidopsis* and tomato groupIII WRKY proteins. The trees were constructed using MEGA6.06.

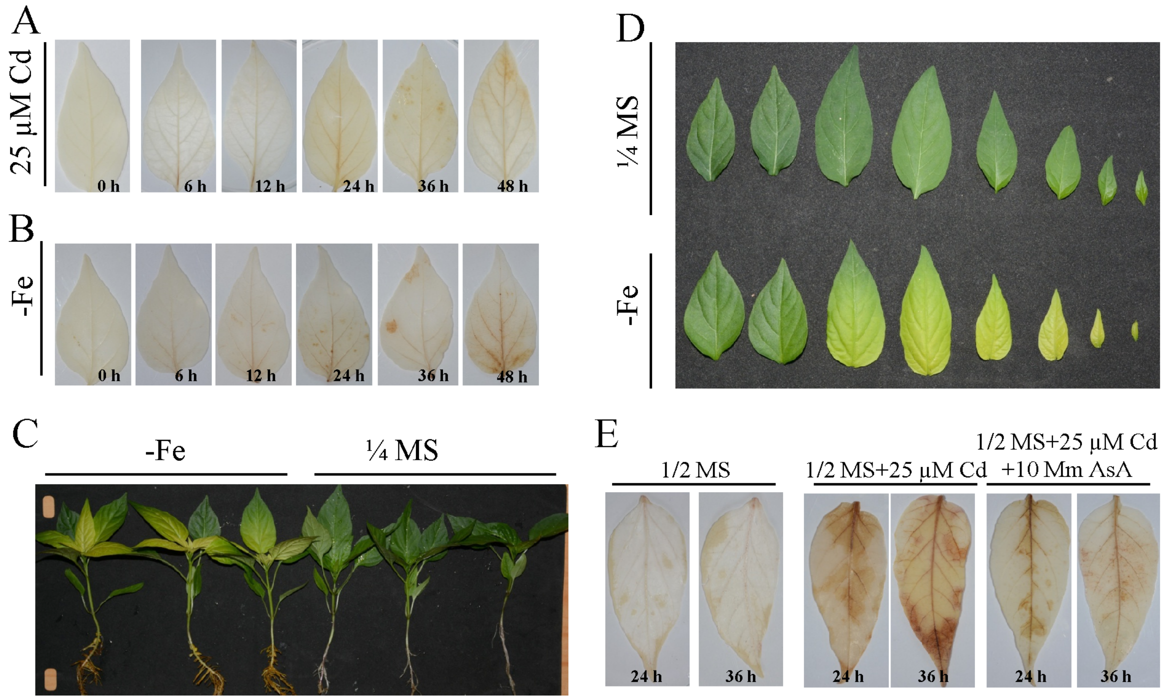

Figure S2. Cd stress and Fe deficiency promotes H_2O_2 accumulation.

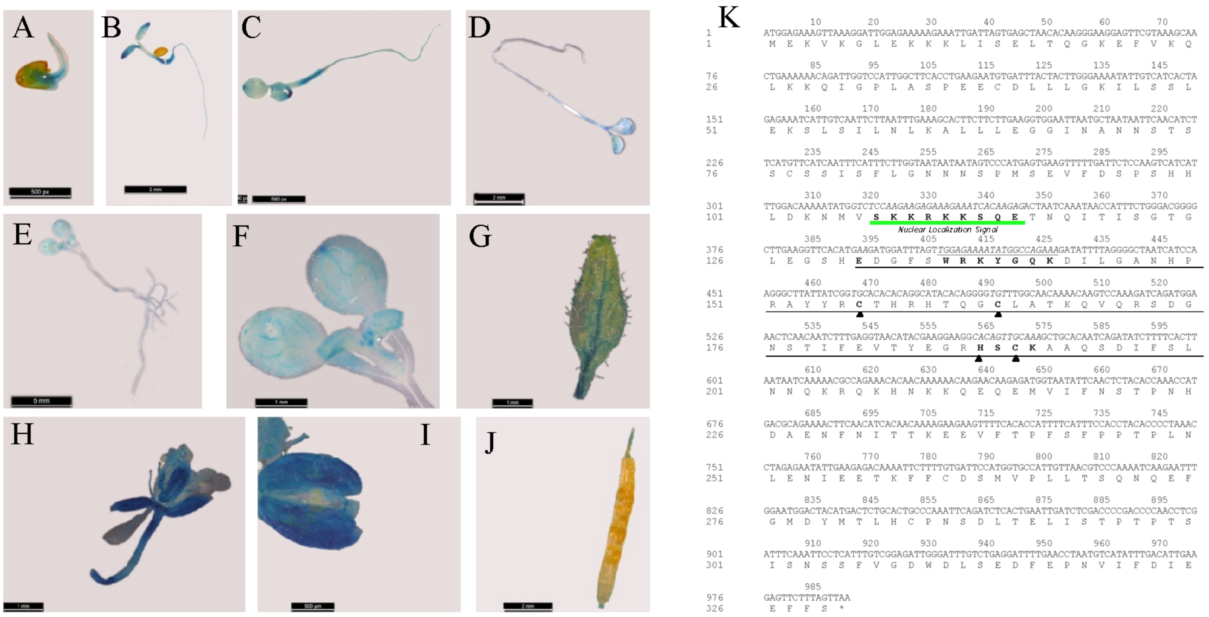

Figure S3. GUS expression in transgenic *pCaWRKY41::GUS Arabidopsis* plants under normal growth conditions.

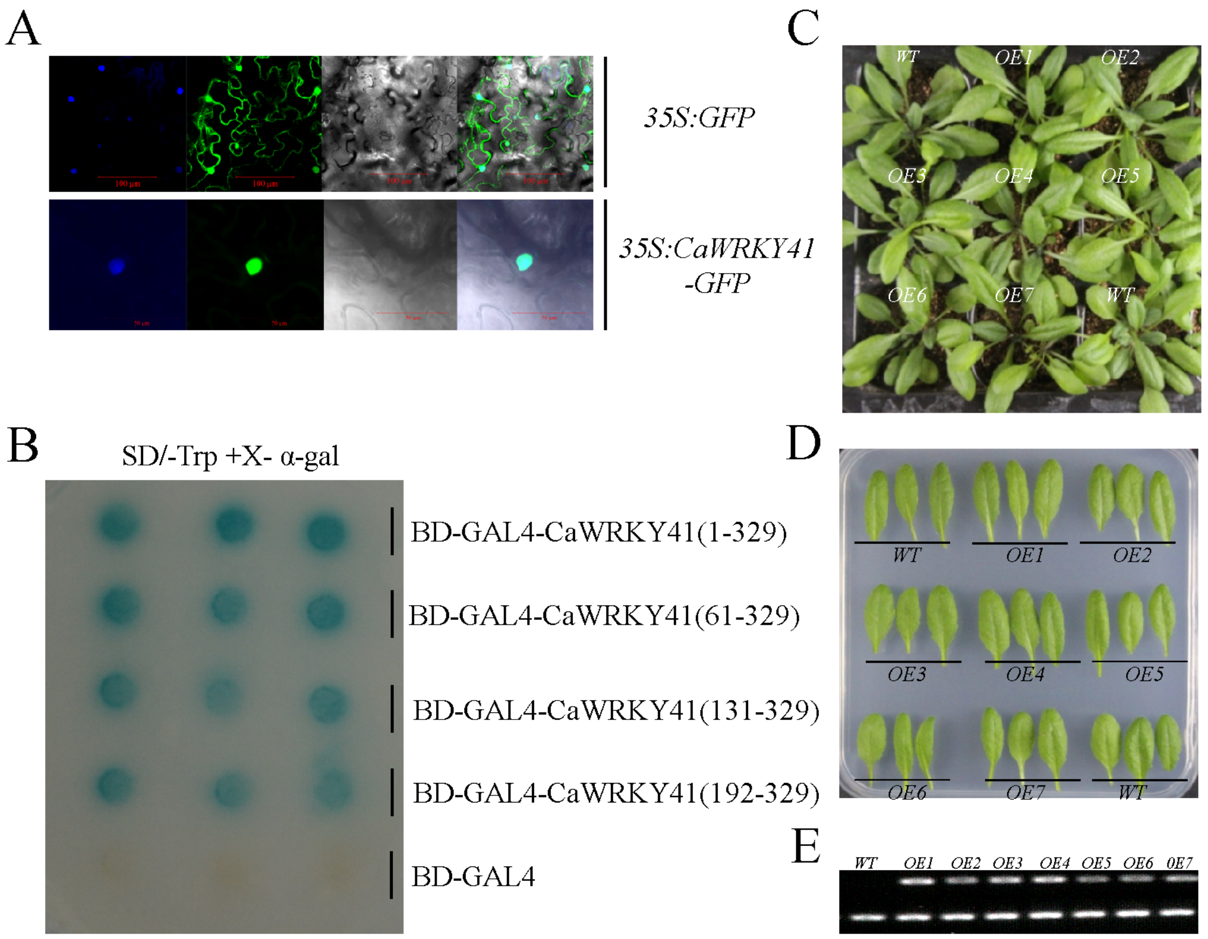

Figure S4. CaWRKY41 is a transcriptional activator localized to the nucleus.

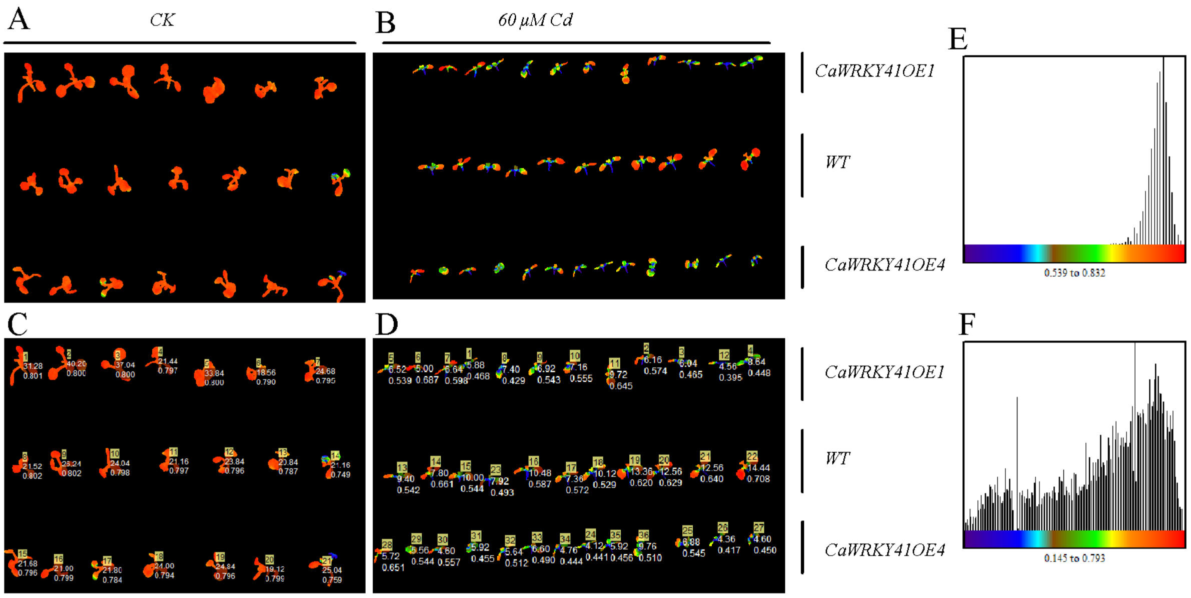

Figure S5. Analysis of the effects of Cd stress on plant growth using chlorophyll fluorescence imaging before the appearance of visual effects on plant growth.

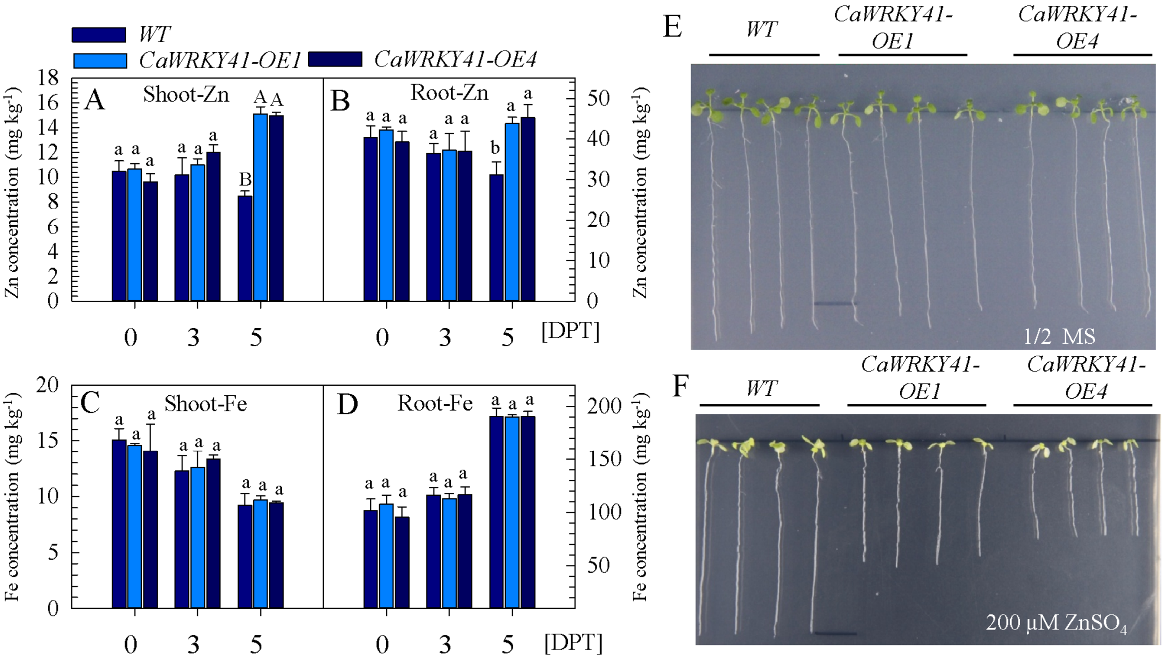

Figure S6. Effect of Cd treatment on Zn concentrations in *Arabidopsis*.

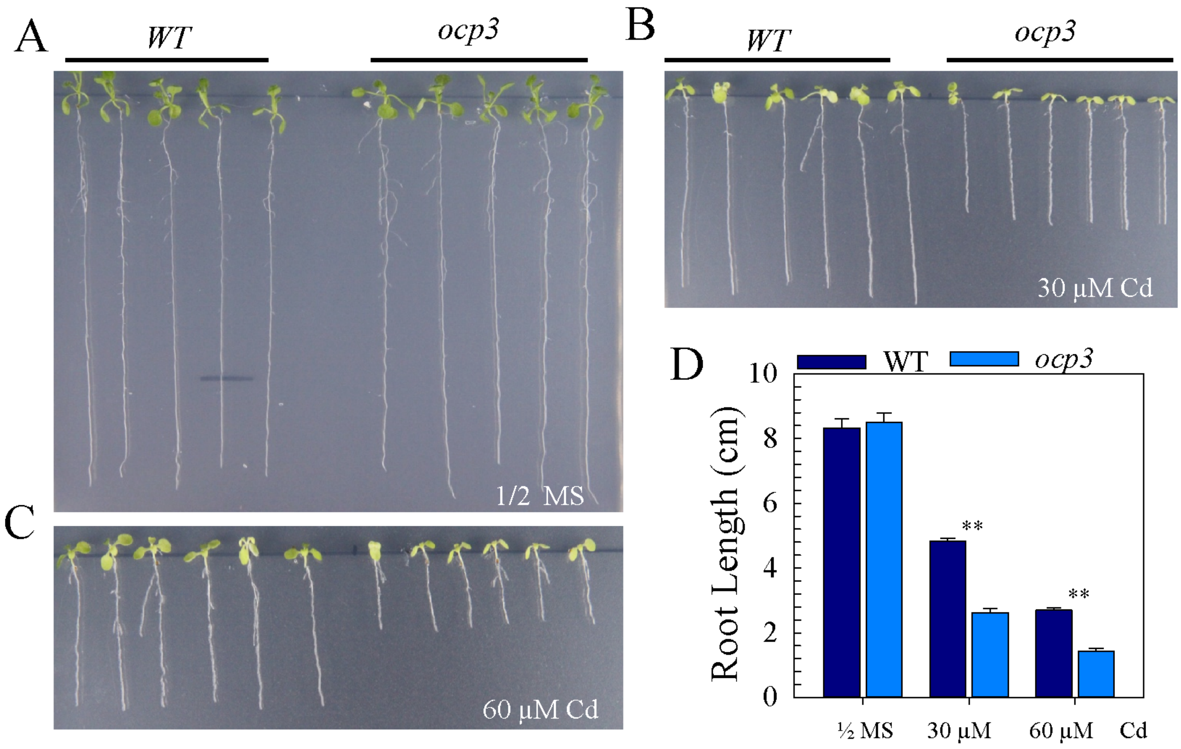

Figure S7. The *Arabidopsis ocp3* (*overexpressor of cationic peroxidase 3*) mutant shows reduced tolerance to Cd stress.

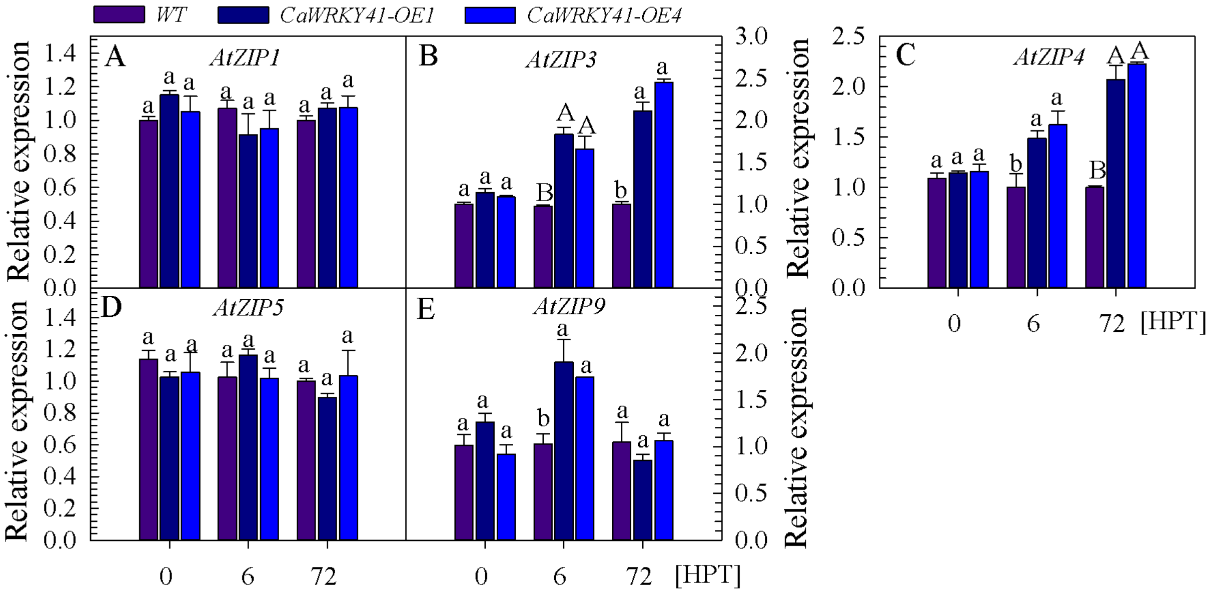

Figure S8. RT-qPCR analysis of the *ZIP* members involved in Zn uptake.

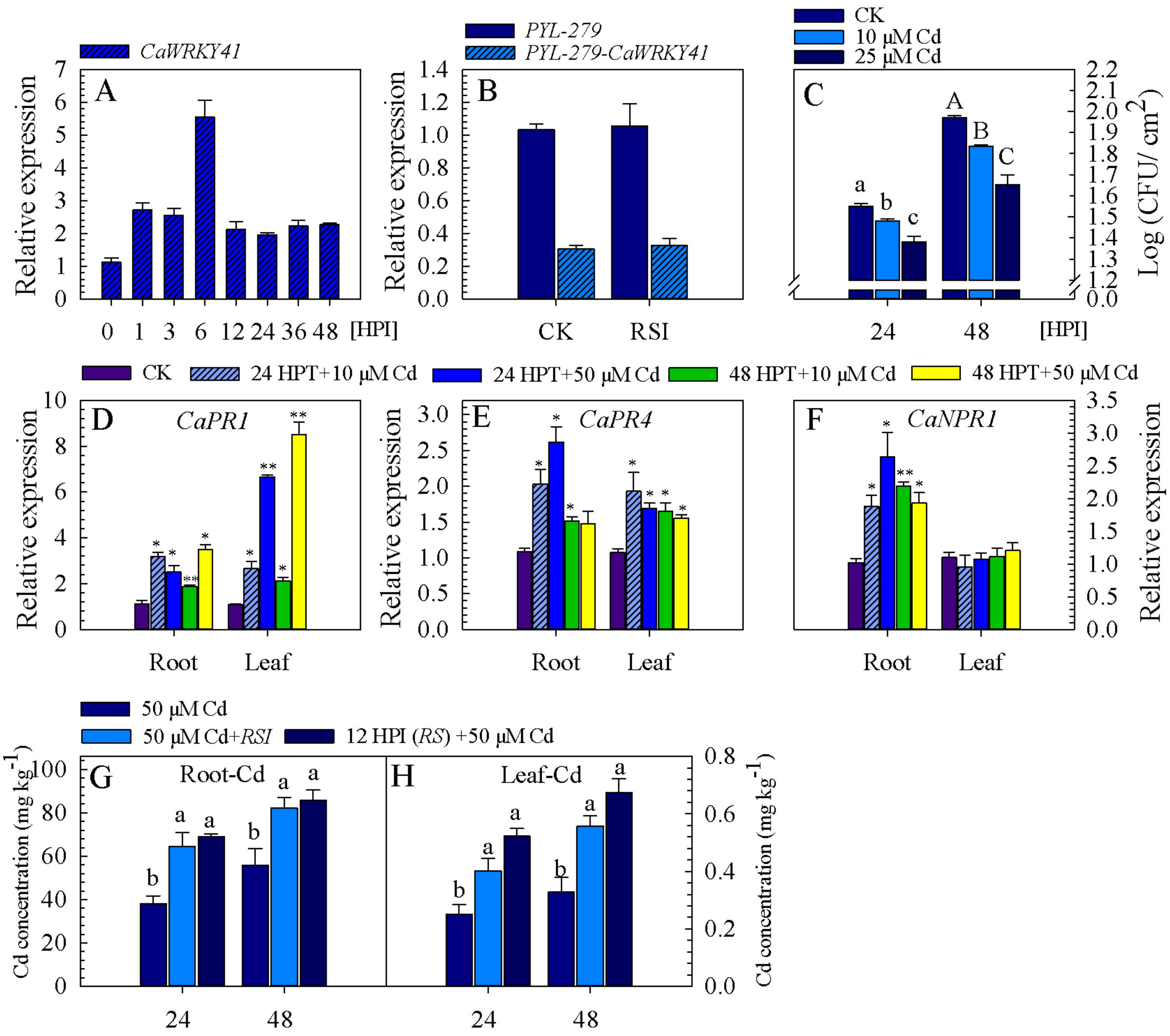

Figure S9. Cd inhibits *Ralstonia solanacearum* growth and *R. solanacearum* infection increases Cd uptake.
